# Identification of distinct HIV reservoir phenotypes and associated immune landscapes

**DOI:** 10.1172/jci.insight.203765

**Published:** 2026-05-28

**Authors:** Ruoyu Wang, Aparna Bhattacharyya, Lily Pohlenz, Erin N. Shirk, Hayley S. Romero, Katherine Haas, Jennifer Coughlin, Raha Dastgheyb, Leah Rubin, Rebecca T. Veenhuis

**Affiliations:** 1 Department of Molecular and Comparative Pathobiology,; 2 Department of Neurology, and; 3 Department of Psychiatry and Behavioral Sciences, Johns Hopkins University School of Medicine, Baltimore, Maryland, USA.; 4 Department of Psychiatry, University of Texas Southwestern Medical Center, Dallas, Texas, USA.; 5 Department of Epidemiology, Johns Hopkins University, Bloomberg School of Public Health, Baltimore, Maryland, USA.

**Keywords:** AIDS/HIV, Immunology, Inflammation, Adaptive immunity, Innate immunity, Monocytes

## Abstract

Virally suppressed people with HIV (PWH) remain at risk for developing comorbidities due to chronic inflammation with one potential contributor being the HIV reservoir. Associations between the CD4 reservoir and inflammation have been extensively characterized, while the role the monocyte reservoir is poorly understood despite evidence that inflammatory monocytes play a role in HIV-associated comorbidities. Additionally, most studies focus on a single cellular reservoir, while it is highly likely that these reservoirs are interdependent. In a cohort of 164 PWH, we used the intact proviral DNA assay to quantify cell-specific reservoirs, applied unsupervised clustering to identify reservoir phenotypes, and then determined if reservoir phenotypes were associated with distinct immune signatures compared with people without HIV. Five unique reservoir clusters emerged, driven primarily by variability in the monocyte reservoir, and each associated with a distinct immune landscape. These included profiles characterized by systemic inflammation, leukocyte–vascular activation, T cell activation with vascular and neuronal injury, enhanced CD8 activation and NK cell recovery, and altered monocyte survival, activation, and migration. This multidimensional approach provides a framework to identify reservoir-immune profiles that may explain heterogeneity in inflammation, despite viral suppression and may inform strategies to mitigate HIV-associated comorbidities.

## Abstract 2

Well-treated people with HIV have different reservoir patterns that are linked to inflammation and may explain varied health risks and help guide tailored treatments.

## Graphical abstract

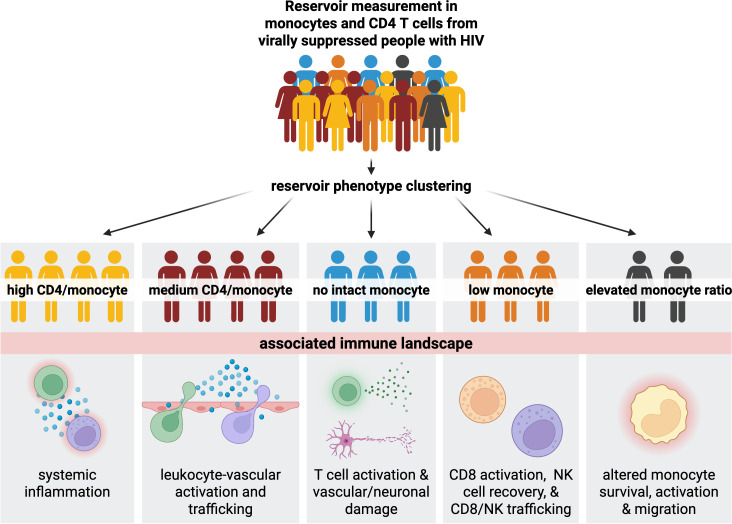

## Introduction

More than 4 decades since its discovery, HIV continues to pose a significant global health burden. Despite the success of antiretroviral therapy (ART) in achieving durable suppression of plasma viremia, people with HIV (PWH) continue to remain at risk for developing non-AIDS–related comorbidities such as cognitive impairment, cardiovascular disease (CVD), and liver disease (1, 2). A hallmark of these conditions is chronic immune activation and inflammation, which persist even under long-term ART and predict adverse clinical outcomes (3). The precise drivers of this immune dysfunction are not yet fully understood, with a potential contributor being the HIV reservoir, a pool of latently infected cells that evades immune clearance and persists with an exceptionally long half-life (4–7).

Historically, reservoir measurements have focused on total HIV DNA within peripheral blood mononuclear cells (PBMCs), which is cost effective and only requires small amounts of blood (8). However, a major limitation of such bulk measurements is that it does not delineate the quality of the integrated HIV genomes. The development of the Intact Proviral DNA Assay (IPDA) and related droplet digital PCR (ddPCR) approaches has resulted in the ability distinguish intact from defective proviruses with a greater sensitivity, allowing for precise quantification of low levels of proviral DNA in a high-throughput fashion (9). This approach has been used to study multiple cellular reservoirs, and previous work has demonstrated that both myeloid (monocytes and macrophages) and CD4 T cells comprise the latent HIV reservoir (10–14). Despite the flexibility of the IPDA approach, most studies only focus on the HIV reservoir in CD4 T cells or monocytes in isolation. However, there is a strong likelihood that both cellular reservoirs are interdependent and influence chronic inflammation throughout ART in a concurrent fashion.

The CD4 T cell reservoir, and its corresponding associations with chronic inflammation, have been extensively characterized. Increased immune exhaustion (PD-1, LAG-3) and activation (CD38, HLA-DR) on CD4s are associated with larger reservoirs (15, 16) as well as lower CD4 T cell nadir, CD4/CD8 ratio, and CD4 T cell counts (17). However, the role of the monocyte reservoir in shaping immune activation and HIV-associated comorbidities remains poorly understood. The monocyte reservoir, like CD4 T cells, can be measured in the blood and provides insights into myeloid reservoir dynamics. Monocytes, short-lived circulating cells that migrate into tissues and differentiate into long-lived macrophages, contribute to chronic inflammation via the release of proinflammatory cytokines and chemokines (18). HIV infection results in increased proportions of CD16^+^ monocyte subsets, including intermediate and nonclassical monocytes, which are associated with disease progression and highly susceptible to HIV infection (19–22). CD16^+^ monocytes remain elevated despite effective ART; they are associated with aberrant inflammation and immune dysfunction and reported to house the majority of the monocyte reservoir (22–24). In addition, plasma levels of soluble CD163 (sCD163), sCD14, TNF, C-reactive protein (CRP), and IL-6, immune analytes secreted by inflammatory monocytes or known to activate these cells, remain elevated despite long-term ART, suggesting persistent monocyte activation (25–27).

Prolonged activation of CD4 T cells and monocytes is a powerful predictor of morbidity and mortality during ART (28). Specifically, the decline of CD4 T cells is associated with increased risk of CVD, cancer, and death among virally suppressed PWH (29), while inflammatory CD14^+^/CD16^+^ intermediate monocytes serve as an independent predictor for CVD (28) and have been shown to have enhanced migratory capacity across the blood-brain barrier, contributing to ongoing CNS infection and neuroinflammation (30, 31). Importantly, increased inflammatory monocyte proportions, HIV DNA in monocytes, and myeloid activation markers (sCD163, sCD14) have been associated with CVD (32), cognitive impairment (11, 33–36), and liver disease (37) in PWH.

Collectively, these observations highlight the need to determine the role the HIV reservoir in both cell types may play in persistent chronic inflammation. No study to date has examined variation in reservoir size and phenotypic distributions across multiple cell types to determine (a) if there are clusters of PWH with similar reservoir patterns and (b) if reservoir phenotypes may elucidate unique inflammatory signatures that can be targeted for immune recovery. Therefore, in this study, we aimed to characterize reservoir phenotypes across CD4 T cells and monocytes from virally suppressed PWH. We quantified intact and defective proviruses using the IPDA and applied unsupervised clustering to identify unique reservoir phenotypes. We then evaluated if specific reservoir phenotypes were associated with unique immune landscapes compared with people without HIV (PWoH). This multidimensional approach provides a framework to identify reservoir-immune profiles that may explain heterogeneity in inflammation despite effective viral suppression and help to guide future treatment strategies for HIV-associated comorbidities.

## Results

### Cohort characteristics.

In total, 164 PWH and 63 PWoH were included in this study and recruited from Baltimore, Maryland, USA, and the surrounding areas (Table 1 and Supplemental Table 1; supplemental material available online with this article; https://doi.org/10.1172/jci.insight.203765DS1). On average, PWH were 58 years of age (SD = 10; range = 29–80), 65% Black, 40% male, and 13 years of education (SD = 3, range 7–26). All PWH were on active ART, for a median of 22 years for those with data available (SD = 8.6, range = 1–40, 27% unknown), with a median CD4 count of 743.5 cells/μL and 85% undetectable viremia (<20 copies/mL); 98% had a viral load of < 75 copies/mL. Median detectable viral loads were 35.5 copies/mL, with 4 participants (2.4%) that had high levels of viremia despite actively taking ART (range = 3,260–52,200 copies/mL). These participants were included in the reservoir clustering analysis but omitted from all immune related analyses. Lifetime mental health and substance history were also assessed. Using the Structured Clinical Interview, for DSM-5 (SCID-V), 44.5% of PWH met criteria for lifetime major depressive disorder (MDD), 16% posttraumatic stress disorder (PTSD), and 33.5% for anxiety disorders. Substance use was characterized as lifetime use (not disorder), with 25% reporting alcohol use, 53% tobacco, 56% marijuana, 36% cocaine, and 30.5% opioid use. All PWH had PBMCs available for reservoir analysis, 88% had basic immunophenotyping, 56% had in-depth immunophenotyping, and 52% had soluble immune analyte data available for analysis.

On average, PWoH were 63 years of age (SD = 13.4, range = 30–80), 44% Black, 65% male, and 14 years of education (SD = 3.8, range = 8–26). Compared with PWH, PWoH reported significantly lower rates of lifetime MDD (17%, *P* < 0.0002), tobacco (31%, *P* < 0.01), marijuana (25%, *P* < 0.0001), cocaine (21.5%, *P* < 0.05), and opioid use (14%, *P* < 0.01). However, rates of lifetime PTSD (14%, *P* = 0.85), anxiety (31%, *P* > 0.9), and alcohol use (18.5%, *P* = 0.37) were similar (Supplemental Figure 1, A and B). All PWoH had basic and in-depth immunophenotyping data, and 76% had immune analyte data available for analysis.

### Paired reservoir measurements demonstrate that the HIV reservoir is heterogenous.

**Figure 1:**
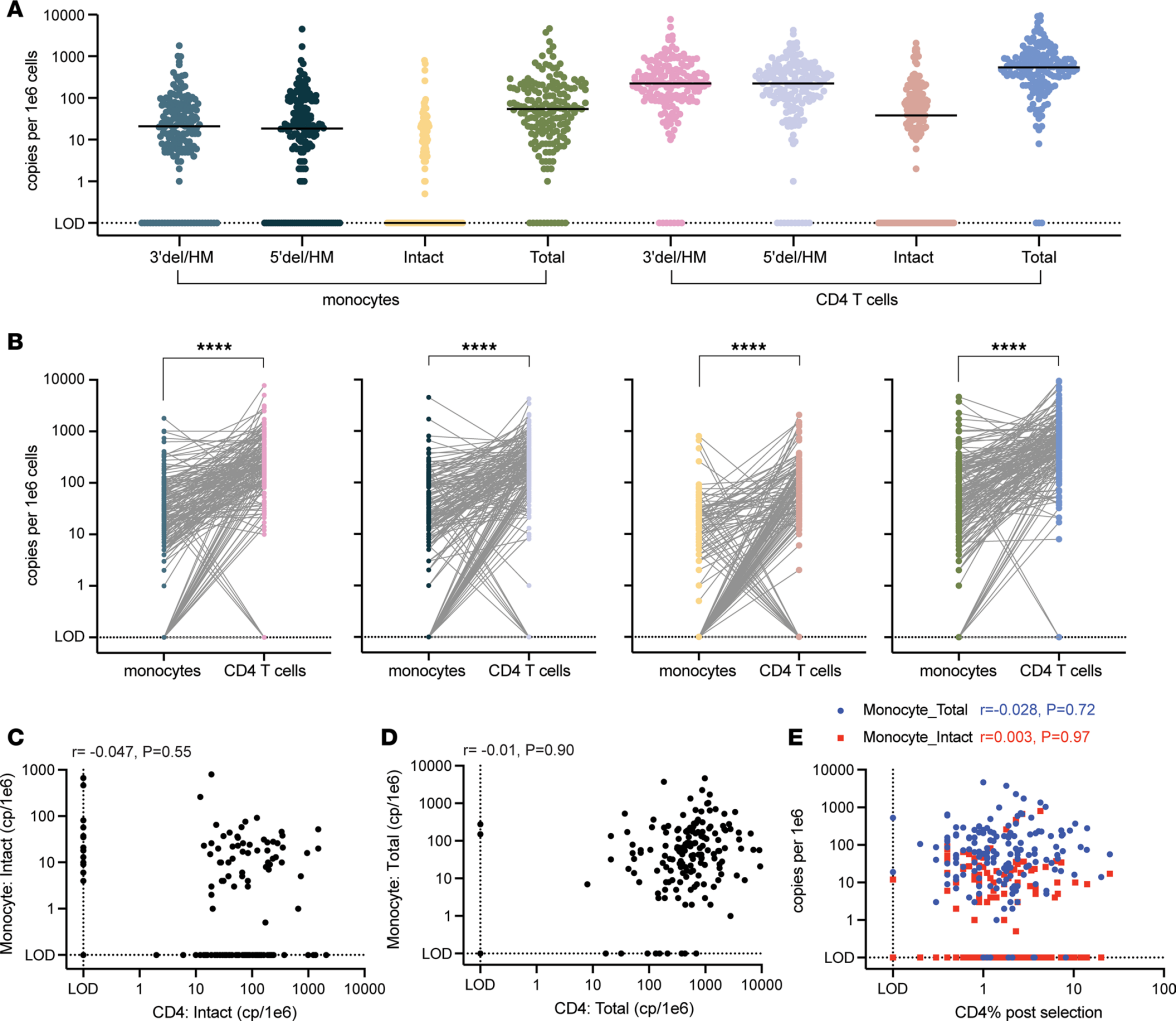
Paired reservoir measurements demonstrate that the HIV reservoir is heterogenous. (**A**) IPDA was used to measure HIV 3′ and 5′ defective (del/HM), intact and total genomes in monocytes and CD4 T cells from 164 people with HIV (PWH). (**B**) Comparing HIV reservoir measures between paired monocytes and CD4 T cells, **** *P* < 0.0001, Wilcoxon matched-pairs signed rank test. (**C**–**E**) Associations between intact monocyte signal and intact CD4 T cell signal, monocyte and CD4 total, and monocyte total (blue) and monocyte intact (red) with CD4% post selection. Pearson correlation *r* and *P* values are displayed with graphs (**C**–**E**).

Building on our previous studies (10, 38), we assessed the quality and quantity of HIV DNA genomes in monocytes and CD4 T cells isolated from the same blood draw in 164 PWH, which is the largest sample size to date to assess both cellular reservoirs (Figure 1A and Table 1). TLR2 selection was used to isolate monocytes as this marker has been shown to efficiently isolate all subsets with minimal contamination from other cell types (10, 11, 39) (Supplemental Figure 2, A and B). Overall, our monocyte selections had a median purity of 92% (SD = 10.3) TLR2^+^ cells with a median of 1.7% CD4 T cell contamination (SD = 3.3; Supplemental Figure 2C and Supplemental Table 2), which is a similar efficiency reported by FACS methodologies. Additionally, a higher median number of TLR2^+^ cells were assessed compared with CD4 T cells (318,000 versus 136,000), as the HIV reservoir is typically present at a lower level in monocytes (Supplemental Figure 2D). Comparable with our previous reports, we observed that 94% of participants had detectable provirus in monocytes (median of detectable provirus [mdn-dp] 62 copies [cp]/1 × 10^6^ cells) in at least 1 form, 42% had detectable intact (mdn-dp 18 cp/1 × 10^6^ cells), 80% had detectable 5′ deleted/hyper mutated ([del/HM] mdn-dp 32 cp/1 × 10^6^ cells) ,and 86% had detectable 3’ del/HM (mdn-dp 30 cp/1 × 10^6^ cells) proviruses. In CD4 T cells, 99% of participants had detectable provirus in CD4s (mdn 549 cp/1 × 10^6^ cells), 72% had detectable intact (mdn-dp 72 cp/1 × 10^6^ cells), 95% had detectable 5’del/HM (mdn-dp 252 cp/1 × 10^6^ cells), and 96% had detectable 3′ del/HM (mdn-dp 241 cp/1 × 10^6^ cells) provirus. On average, the monocyte reservoir was present at approximately 1 log lower that the CD4 reservoir in all genome categories. When comparing paired reservoir measures between cell types, we observed that there were multiple patterns within each genome category (Figure 1B). A large proportion of participants had undetectable monocyte reservoirs with large CD4 reservoirs, while others had similar sized reservoirs in both cell types, and a fraction had larger monocyte reservoirs compared with CD4s. Additionally, we compared paired monocyte and CD4 T cell intact and total measurements to determine if the monocyte signal observed could have been contributed by contaminating CD4 T cells. We observed no associations between monocyte and CD4 intact (*r* = –0.047, *P* = 0.55, Figure 1C) or monocyte and CD4 total (*r* = –0.01, *P* = 0.90, Figure 1D). As well as no association between the percent of contaminating CD4 T cells after selection with monocyte intact or total reservoir measures (*r* = –0.028, *P* = 0.72, *r* = 0.003, *P* = 0.97, respectively; Figure 1E). These data demonstrate that HIV reservoir patterns are heterogenous and suggest that there are likely distinct reservoir phenotypes within PWH.

### Cluster analysis demonstrates 5 unique reservoir phenotypes driven by monocyte reservoir characteristics.

To investigate if there are distinct reservoir phenotypes, we implemented a clustering pipeline consisting of dimension reduction and Gaussian Mixture Models using the paired monocyte and CD4 T cell 3′ del/HM, 5′ del/HM and intact variables. Dimension reduction was accomplished on log transformed and scaled data using Principal Components Analysis (PCA) with varimax rotation to reduce the number of variables and determine the relationships within the IPDA variables. We observed that a 3-component solution explained at least 80% of the variability (Supplemental Figure 3A). The 3 components reflected CD4 reservoir (RC1: CD4_intact, CD4_5′ del/HM, CD4_3′ del/HM), defective monocyte reservoir (RC2: TLR2_5′ del/HM, TLR2_3′ del/HM), and intact monocyte reservoir (RC3: TLR2_intact, Supplemental Figure 3B). Gaussian Mixture Models were fit to the retained components. Models ranging from 2 to 10 clusters across multiple covariance structures were evaluated, and it was found that a 5-cluster solution demonstrated optimal fit across the models with an Akaike Information Criterion (AIC) of 1219.08, Bayesian Information Criterion (BIC) of 1324.475, LogLik of –575.54 and an Entropy of 0.851 (Supplemental Figure 3C). Posterior membership probabilities were extracted, and a 75% probability threshold was applied to retain high-confidence cluster assignments (Supplemental Figure 3D), yielding a final analytic sample of 151 participants. This threshold, balanced cluster purity with sample retention, and excluded participants did not differ significantly from retained participants in demographic or clinical characteristics (Table 1). To assess whether the specific value assigned to data points that were below the limit of detection (LOD) of the IPDA influenced our findings, we performed a sensitivity analysis. We compared our primary Gaussian Mixture Model solution against 4 assignment values: 0.05, 0.01 (used in original analysis), 0.001, and 0. Overall, the clustering results were exceptionally stable regardless of the value assigned to undetectable data points. The Adjusted Rand Index (ARI) values remained above 0.96 for all comparisons, indicating nearly identical participant assignments as a score of 1.0 represents perfect agreement between 2 models, while a score of 0 indicates agreement no better than random assignment (Supplemental Figure 3, E and F). These data demonstrate that, while detection frequency contributes to cluster separation, the cluster separation is driven by robust biological signals rather than artifacts of how undetectable values were assigned.

**Figure 2:**
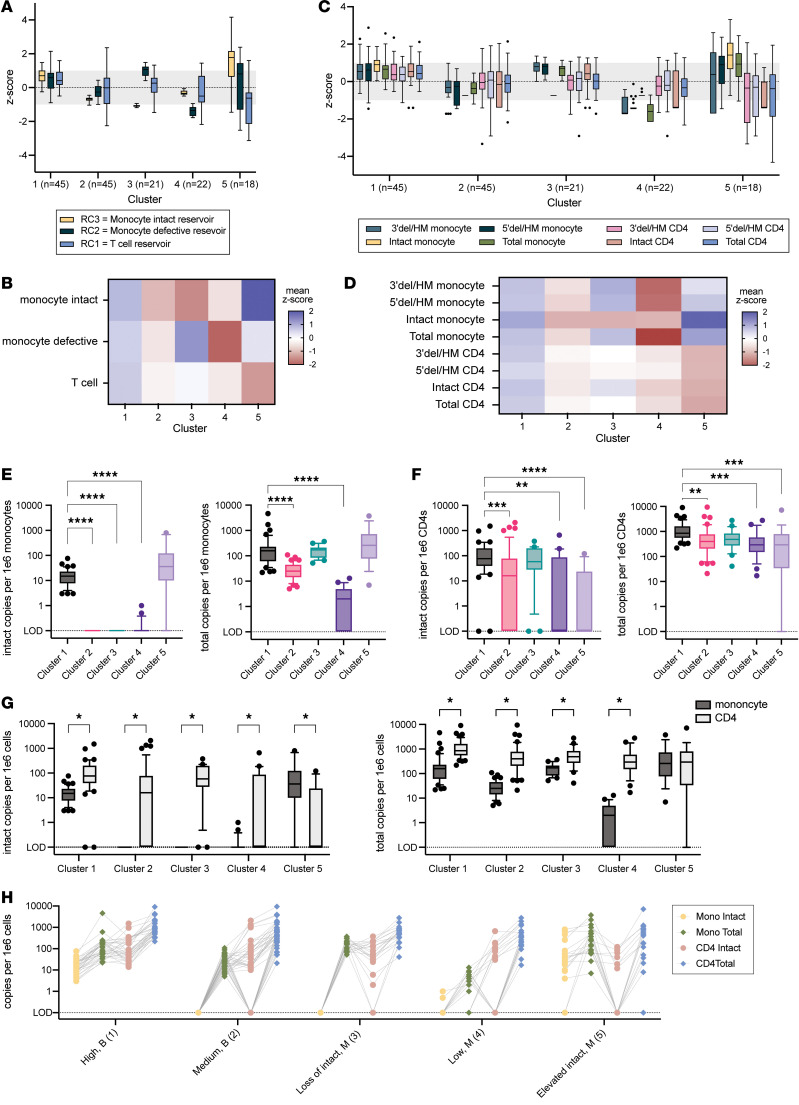
Five unique reservoir phenotypes primarily driven by monocyte reservoir characteristics. (**A** and **B**) *Z* score of PC components within each defined reservoir cluster (**A**) and their corresponding heatmap displaying mean *z* scores (**B**). (**C** and **D**) *Z* scores of individual variables within each defined cluster (**C**) and the corresponding heatmap displaying mean *z* scores (**D**). (**E** and **F**) Comparison of absolute intact and total genome values across clusters within monocytes (**E**) and CD4 T cells (**F**). Statistics were completed using Kruskal-Wallis test with a Dunn’s correction for multiple comparisons. (**G**) Comparison of intact and total genome copies between monocytes and CD4 T cells within clusters, statistics were completed using multiple Mann-Whitney *U* tests with a correction for multiple comparisons using a 2-stage step-up FDR of 5%. (**H**) Patterns of paired intact and total copies per million cells in monocytes and CD4 T cells for each reservoir phenotype. Sample size for Cluster 1 *n* = 45, Cluster 2 *n* = 45, Cluster 3 *n* = 21, Cluster 4 *n* = 22, Cluster 5 *n* = 18, box and whiskers plots show 10–90 percentile, dots are data points outside of that range (**A**, **C**, and **E**–**G**). *P < 0.05, **P < 0.01, ***P < 0.001, ****P < 0.0001.

Overall, the clustering analysis yielded 5 unique phenotypes (Figure 2, A–D), with 30% of PWH associated with Cluster 1 (*n* = 45), 30% Cluster 2 (*n* = 45), 14% Cluster 3 (*n* = 21), 14% Cluster 4 (*n* = 22), and 12% Cluster 5 (*n* = 18). Cluster 1 showed high monocyte and CD4 T cell reservoir levels, whereas Cluster 2 showed medium monocyte and CD4 T cell reservoirs. Cluster 3 was characterized by abundant defective monocyte and intermediate CD4 T cell reservoirs but little to no intact monocyte reservoir. Cluster 4 demonstrated low monocyte reservoir levels and intermediate CD4 T cell reservoirs. Cluster 5 displayed high monocyte but comparatively low CD4 T cell reservoirs and showed the greatest within-cluster variability. Overall, there were no significant differences in the demographics (sex, age, race, education), CD4 count, years on ART, lifetime mental health, or SUD reported in the participants assigned to each cluster (Supplemental Table 1 and Supplemental Figure 1, C and D).

We used the intact and total genome copies per million cells to describe patterns across clusters and cell types and determine the appropriate labels for the reservoir phenotypes (Figure 2, E–G). The total genome measure was used in place of the 3′ del/HM and 5′ del/HM measurements as it encompasses all reservoir measures, and because the 3′ del/HM and 5′ del/HM measures had similar patterns to the total measure (Supplemental Figure 4, A and B). As Cluster 1 had minimal variability and the highest reservoir in both cell types, we assigned this as the referent cluster to which we compared the level of intact and total reservoirs across clusters in monocytes (Figure 2E) and CD4 T cells (Figure 2F). We observed that the monocyte intact reservoir was significantly higher in Cluster 1 compared with clusters 2, 3, and 4 (all *P* < 0.0001), but there was no significant difference with Cluster 5. Additionally, we observed that the total monocyte reservoir was significantly higher in Cluster 1 compared with clusters 2 and 4 (all *P* < 0.0001), but no significant difference with clusters 3 and 5. Differences in monocyte patterns did not appear to be due to cell number assessed in the IPDA, as clusters 1, 2, 4, and 5 had similar cellular inputs. Only Cluster 3 differed and was significantly lower than clusters 2 and 4 (Supplemental Figure 3E). However, despite lower cellular input, Cluster 3 had equivalent levels of total and defective HIV genomes compared with Cluster 1. In CD4 T cells, we observed that Cluster 1 had significantly higher intact and total genomes compared with clusters 2, 4, and 5 (all *P* < 0.001) but no significant difference with Cluster 3 in either genome type. Differences in CD4 patterns were not due to cell number assessed, as all clusters had similar cellular inputs (Supplemental Figure 4, E and F). Additional comparisons between clusters and genome categories, demonstrating high variability in the monocyte reservoir with limited variability in the CD4 reservoir, are reported in Supplemental Figure 4G.

When comparing the size of the intact and total reservoirs between cell types within clusters, we observed that, in clusters 1–4, CD4 T cells had significantly higher levels of both intact and total genomes (all *P* < 0.05). However, Cluster 5 was a unique cluster in which monocytes had significantly higher levels of intact genomes compared with paired CD4 T cells (*P* < 0.05), with no difference in the total or defective reservoirs (Figure 2G and Supplemental Figure 4, C and D). Overall, there was greater variability in the monocyte reservoir phenotypes compared with the CD4 reservoir phenotypes across clusters; therefore, the changes in the monocyte reservoir were predominately used to label the clusters, with Cluster 1 indicating high reservoir in both cell types (High, both [B]), Cluster 2 indicating medium reservoir in both cell types (Medium, B), Cluster 3 indicating specific loss of monocyte intact (Loss of intact, monocyte [M]), Cluster 4 indicating low reservoir in monocytes (Low, M), and Cluster 5 indicating elevated monocyte intact reservoir (Elevated intact, M; Figure 2H).

### Unique cellular and soluble immune landscapes are associated with each reservoir phenotype compared with PWoH.

We sought to determine if there were unique immune landscapes associated with reservoir phenotypes compared with PWoH, as an overarching goal of understanding chronic inflammation in PWH would be to develop precision medicine approaches to target specific inflammatory patterns in distinct clusters of PWH. Therefore, we assessed basic immunophenotyping (including CD4 and CD8 T cells, classical, intermediate and nonclassical monocytes percentages and CD4/CD8 ratios), in-depth immunophenotyping (including T cell memory and activation status, monocytes activation and trafficking phenotypes, NK cell phenotypes and absolute cell numbers), and soluble immune analytes (including general and neuroinflammation, leukocyte recruitment, vascular function and endothelial integrity) for each reservoir cluster and demographically matched PWoH. All analyses are shown as average values scaled to the group of PWoH (*z* scores), indicating deviations relative to PWoH rather than differences within PWH.

**Figure 3:**
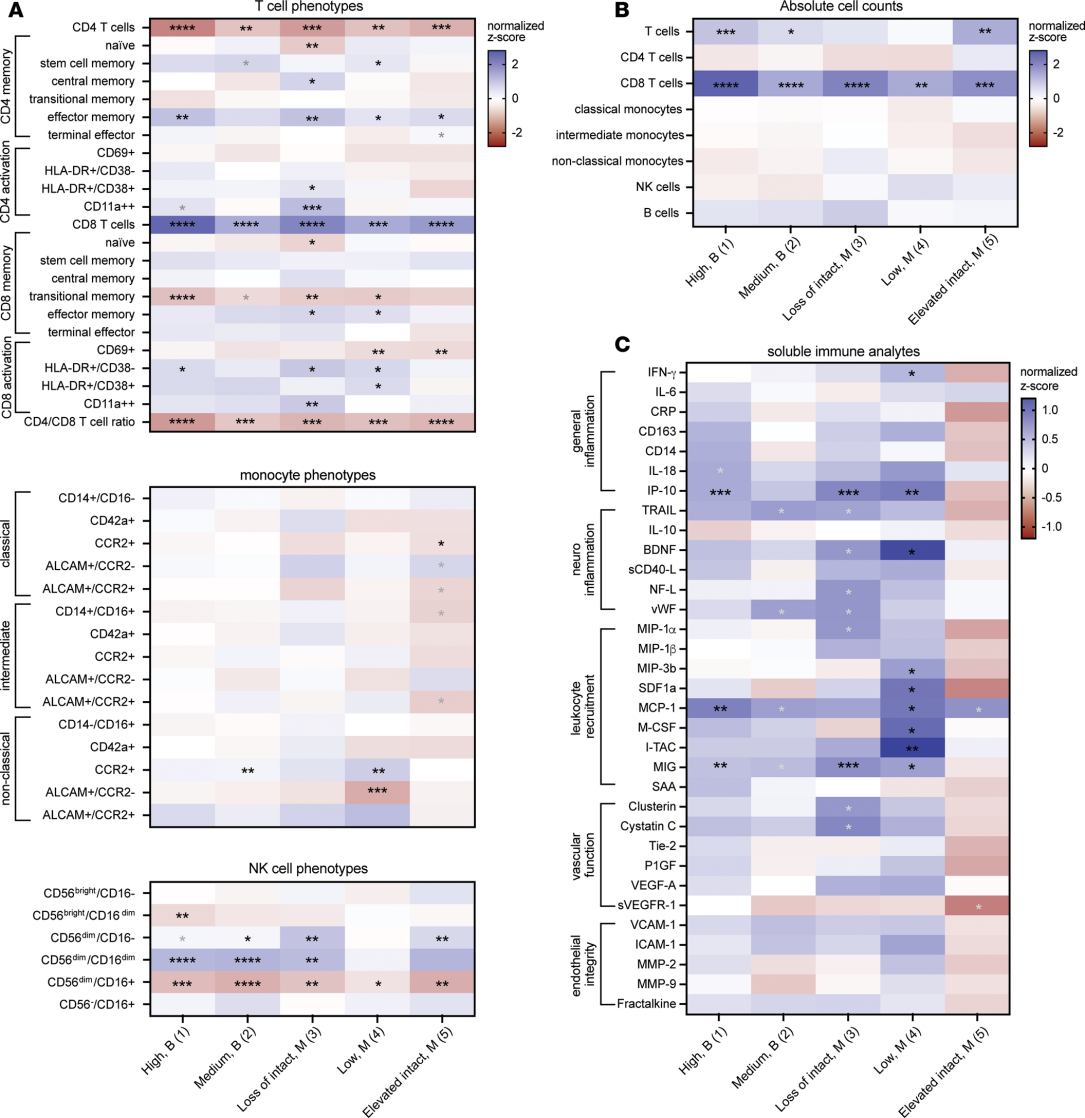
Distinct immune landscapes associated with reservoir phenotype clusters. (**A**–**C**) Average normalized *z* score of PWH compared with PWoH for T cell, monocyte and NK cell phenotypes (**A**), absolute cell counts (cells per μL, **B**) and soluble immune analytes (**C**). Statistics were completed using multiple Mann-Whitney *U* tests with a correction for multiple comparisons using a 2-stage step-up exploratory FDR of 15%, *P < 0.05, **P < 0.01, ***P < 0.001, ****P < 0.0001. Black asterisk indicates significance post-FDR, gray asterisk indicates significance pre-FDR but did not hold post.

Common immunophenotyping signatures observed across the reservoir clusters involved a combination of a significantly lower CD4/CD8 ratio, increased CD4 effector and CD8 transitional memory, increased inflammatory (CD56^dim^/CD16^dim^) and unconventional (CD56^dim^/CD16^–^) NK cells, and decreased mature cytotoxic (CD56^dim^/CD16^+^) NK cells (all *P* < 0.05; Figure 3A and Supplemental Figure 5), suggesting overall lower T cell health and NK cell function as has been previously reported in HIV (15–17). Cluster 1 (high reservoir) primarily demonstrated the shared T/NK cell signature with minimal other markers significantly altered, suggesting that T and NK cells are the primary populations perturbed in this group. Cluster 2 (medium reservoir) demonstrated increased percentages of inducible nonclassical monocytes (iNCM, CD14^–^/CD16^+^/CCR2^+^) and the shared T/NK cell signature, suggesting alterations in T cell, monocyte, and NK cell populations. Cluster 3 (loss of intact myeloid reservoir) maintained the shared NK cell phenotype and demonstrated the strongest CD4 and CD8 memory signature, with significantly fewer naive CD4s and CD8s (all *P* < 0.01) and significantly higher CD4 and CD8 effector memory (all *P* < 0.05) and CD4 central memory percentages (*P* < 0.05). Cluster 3 also had a strong CD4 and CD8 activation signature with significantly higher CD11a^++^ and HLA-DR^+^ percentages (all *P* < 0.05), suggesting that T cell function is more extensively perturbed in this cluster. Cluster 4 (low myeloid reservoir) demonstrated the shared T cell signature, observed in clusters 1 and 2, with an additional CD8 T cell activation signature demonstrating elevated late activation (HLA-DR^+^/CD38^–^ and HLA-DR^+^/CD38^+^) but lower early activation (CD69^+^) marker expression (all *P* < 0.05). The strongest increase in iNCM signal was observed in this cluster as well as a unique recovery of NK cell phenotypes, suggesting alterations to NK cell and CD8 T cell function in this cluster (all *P* < 0.05). Cluster 5 (elevated intact myeloid reservoir) demonstrated a unique monocyte signature with lower percentages of CCR2^+^ and ALCAM^+^/CCR2^+^ classical monocytes and CCR2^+^/ALCAM^+^ intermediate monocytes, suggesting an alteration in the survival and/or trafficking of monocytes in this cluster (all *P* < 0.05). Of note, except for CCR2^+^ classical monocytes, the signature associated with Cluster 5 was one of the few that did not hold after FDR correction. As Cluster 5 was the smallest cluster (*n* = 18), this may be due to sample size and should be further investigated. Absolute cell count signatures observed across all clusters involved higher numbers of CD8 T cells, while higher counts of total T cells were only observed in clusters 1, 2, and 5 (all *P* < 0.05; Figure 3B and Supplemental Figure 5). To determine if there were unique cellular phenotypes between reservoir clusters, we completed additional comparisons and significant findings are highlighted in Supplemental Figure 6 and Supplemental Table 3. These comparisons are not FDR adjusted and intended for exploratory analysis only. However, they demonstrate heterogenous patterns of cellular phenotypes within clusters of PWH across all cell types, with the greatest differences in monocyte phenotypes observed when comparing Cluster 5, while CD4 phenotypes were varied across most clusters, with the exception of a specific naive CD4 signal associated with Cluster 3.

Common soluble immune analyte signatures observed across most clusters involved increased levels of IP-10, MIG, and MCP-1, all of which have been previously shown to increase in virally suppressed PWH (40–43) (Figure 3C and Supplemental Figure 7). Cluster 1 (high reservoir) primarily demonstrated the shared signature with additional elevations in IL-18. Cluster 2 (medium reservoir) demonstrated a signature that involved leukocyte recruitment (MCP-1, MIG), vascular (von Willebrand factor [vWF]), and apoptotic/immune regulatory signaling (TRAIL). Cluster 3 (loss of intact myeloid reservoir) demonstrated a signature of increased leukocyte activation and recruitment (IP-10, MIG, MIP-1α), lymphocyte survival and proliferation (BDNF, TRAIL), and vascular/neurodegenerative stress (vWF, clusterin, cystatin C, NF-L). Cluster 4 (low myeloid reservoir) demonstrated the strongest signature of leukocyte recruitment (IP-10, I-TAC, MIG, MIP-3β), tissue repair (SDF-1α), lymphocyte survival and proliferation (BDNF), and myeloid recruitment/development (MCP-1, M-CSF). Cluster 5 (elevated intact myeloid reservoir) demonstrated a signature of increased monocyte chemotaxis (MCP-1) and increased vascular permeability (sVEGFR-1). To determine if there were unique soluble immune analyte patterns between reservoir clusters, we completed additional comparisons, and significant findings are highlighted in Supplemental Figure 8 and Supplemental Table 3. As mentioned above, these comparisons are not FDR adjusted and are intended for exploratory analysis only. However, they demonstrate heterogenous patterns of immune analytes within clusters of PWH, with most changes occurring in the inflammatory and leukocyte recruitment markers.

Overall, the soluble immune analyte signatures in combination with the cellular signatures suggest reservoir phenotype specific immune landscapes, such as Cluster 1 (high, B) indicating systemic inflammation; Cluster 2 (medium, B) indicating leukocyte-vascular activation and trafficking; Cluster 3 (loss of intact, M) indicating T cell activation associated with vascular and neuronal damage; Cluster 4 (low, M) indicating monocyte induced increase in CD8 activation and NK cell recovery and trafficking; and Cluster 5 (elevated intact, M) indicating altered monocyte survival, activation, and migration (Table 2).

## Discussion

In this study, we identified 5 distinct HIV reservoir phenotypes defined by the quantification of intact and defective proviral genomes within monocytes and CD4 T cells from a large cohort of virally suppressed PWH. These reservoir phenotypes corresponded to unique immune landscapes, demonstrating that heterogeneity in reservoir composition, particularly within monocytes, correlated with distinct immunophenotyping and soluble immune analyte signatures compared with PWoH. This multidimensional approach identified unique reservoir-immune profiles which may contribute to heterogeneity in inflammation despite effective viral suppression and is the first step in identifying specific immune pathways that may be targeted in subgroups of PWH to bring inflammation down to the levels observed in PWoH.

Two overarching reservoir phenotypes emerged: (a) dual-reservoir phenotype, where both monocytes and CD4 T cells harbored elevated (Cluster 1) or intermediate (Cluster 2) total proviral levels, and (b) monocyte-centric phenotypes, where monocyte reservoirs were disproportionately altered (clusters 3 [high defective], 4 [low overall], and 5 [elevated intact]). Reservoir clustering was heavily driven by the monocyte reservoir, as the CD4 reservoir had minimal variability between clusters, with the exception of being significantly higher in Cluster 1. The 2 most common clusters were the “high” (a) and “medium” (b) clusters, containing approximately 60% of the cohort. This suggests that the majority of PWH have detectable reservoirs in both cell types that were proportional to each other. In contrast, the monocyte-centric phenotypes made up 40% of the cohort and demonstrate that, in many PWH, the size of the monocyte reservoir does not necessarily reflect the size of the CD4 reservoir. The “loss of intact” cluster (c) demonstrated that 14% of PWH have high levels of defective provirus in monocytes in the absence of intact. An important subgroup of PWH to investigate as defective proviruses are known to contribute to ongoing inflammation (44). Similarly, the “low” reservoir cluster (d) represents PWH that have low to undetectable total proviral genomes in monocytes and suggest that these individuals may be able to preferentially eliminate or do not form a long-lived monocyte reservoir. The “elevated intact” cluster (e) suggests that a small proportion (~12%) of PWH maintain large monocyte reservoirs, as this was the only cluster in which monocytes harbored significantly higher or equivalent HIV genome levels compared with CD4 T cells. This unique cluster can help elucidate the effect the intact monocyte reservoir may have in shaping immune activation.

To our knowledge, this is the first study of its kind to differentiate reservoir phenotypes by simultaneously assessing the monocyte and CD4 reservoirs, and it is unknown what may have contributed to the distinct patterns in reservoir size in both cell types. As expected, the monocyte reservoir was more difficult to detect compared its CD4 counterpart and required the assessment of significantly more cells to confirm the presence or absence of signal. This is critical, as assessing small numbers of moncoytes will lead to an assumption of the absence of a reservoir and should be considered in future studies aimed at measuring both cell types. Previous work, focused on the reservoir in PBMCs, has suggested that the timing of ART, pretreatment viral load, and chronic inflammation (45–47) play a role in influencing the size of the reservoir, while others argue that the reservoir is not influenced by inflammation (48). Studies that have focused on CD4 T cells have reported similar findings and added additional factors such as clonal expansion, CD4/CD8 ratio, and CD4 T cell function (49–51). Together, these studies suggest the findings observed with PBMCs are likely driven by the CD4 reservoir and unlikely to be representative of variables that alter the size of the monocyte reservoir. However, there are many studies that have demonstrated the inflammatory effect of monocytes in reference to comorbidities (32), and it is possible that the reservoir in this cell type contributes to this systemic inflammation.

We demonstrate that reservoir phenotypes are associated with distinct immune landscapes in PWH compared with PWoH, with the overarching goal of identifying potential immune pathways in subgroups of PWH that could be targeted to treat inflammation. We observed 5 distinct immune landscapes that centered on the major themes of immune cell activation, inflammation, neuro/vascular function, and trafficking patterns. Interestingly, several features were shared across most clusters, including significantly reduced CD4/CD8 ratios (all 5 clusters), also demonstrated by lower percentage of CD4 and higher percentage of and absolute CD8 T cells, lower transitional memory CD8 T cells (clusters 1–4), and significantly elevated effector memory CD4 T cells (clusters 1, 3, 4, 5). NK cell populations were also disrupted in most clusters with a decrease in mature cytotoxic cells (CD56^dim^/CD16^+^, clusters 1–5) and increase in inflammatory (CD56^dim^/CD16^dim^, clusters 1–3), and nonconventional (CD56^dim^/CD16^–^, clusters 1–3, 5) populations. Plasma concentrations of IP-10 (clusters 1, 3, 4), MCP-1 (clusters 1, 2, 4, 5), and MIG (clusters 1, 2, 3, 4) were also significantly elevated. These findings are consistent with previous studies demonstrating low CD4/CD8 ratio (51), disruption of NK cell phenotypes (52, 53), and persistent elevation of IP-10, MCP-1, and MIG in virally suppressed PWH (40–43). While these markers do not differentiate reservoir phenotypes, the consistency of these findings with previous studies provides necessary validation for our method of assessing changes in immune landscapes when comparing PWH to PWoH.

The magnitude and pattern of immune associations varied by reservoir phenotype, suggesting that reservoir heterogeneity may contribute to immune variability during viral suppression when compared with PWoH. Cluster 1 (high reservoir) was associated with the shared immune patterns mention above, as well as elevated CD4 (CD11a^+^) and CD8 (HLA-DR^+^) activation and soluble IL-18. Elevated IL-18, a proinflammatory cytokine produced by the inflammasome, is associated with a variety of inflammatory conditions, including diabetes, atherosclerosis, and kidney and liver disease (54), as well as is a predictor of the efficacy of long-term ART (55). Elevation of this cytokine, in the context of altered T cell and NK function, likely contributes to ongoing systemic immune activation (56) and could be a therapeutic target for PWH with highly persistent dual–cell type reservoirs. Cluster 2 (medium reservoir) demonstrated increased percentages of CCR2^+^ nonclassical monocytes, also termed iNCM, and inflammatory NK cells, in combination with increased concentrations of plasma MIG, MCP-1, TRAIL, and vWF. iNCMs have been shown to play a dual role, they contribute to inflammation and disease by migrating to sites of injury and are involved in resolving inflammation and patrolling the vasculature (57). Additionally, iNCMs have been linked to increased inflammatory NK cells, and are thought to recruit these cells to sites of inflammation, particularly in cancer (58). In PWH increases in iNCMs are associated with cognitive impairment (59). TRAIL and vWF are key biomarkers of neuroinflammation and vascular damage in PWH. TRAIL may have dual effects with increased levels resulting in the attenuation of vascular and T cell inflammation (60), while also promoting neuroinflammation (61). Persistent elevation of vWF, a proinflammatory and prothrombotic factor, contributes to the increased risk of CVD, stroke, neuroinflammation, and other blood clotting complications (62). Together, these cellular and soluble factors in combination with the shared immune signature suggests a neurovascular activation, and trafficking landscape is associated with PWH whom have medium reservoir levels in both cell types compared with PWoH.

Interestingly, the 3 monocyte-centric reservoir phenotypes demonstrated notably unique immune landscapes when compared with PWoH. Cluster 3 (loss of monocyte intact) exhibited the strongest T cell profile with increased CD4 and CD8 activation (CD11a^++^ and HLA-DR^+^) and increased CD4 and CD8 memory subsets (central and effector), with a notable decrease in naive cells. Elevated soluble analytes were associated with immune trafficking (MIP-1α, MIG, IP-10), lymphocyte survival and proliferation (BDNF, TRAIL), and vascular/neurodegenerative stress (vWF, clusterin, cystatin C, NF-L, TRAIL). The increased cellular activation observed in Cluster 3 may be due to high levels of defective proviruses, which generate viral RNAs and proteins, despite ART, and have been shown to contribute to ongoing inflammation through cellular activation and neuroinflammatory processes (63, 64). This is further supported by elevations in NF-L, cystatin C, vWF, and TRAIL as these analytes have all been linked to monocyte activation and cognitive impairment in HIV (11, 30, 36, 61, 62, 65). Therefore, Cluster 3 may represent a phenotype in which high levels of defective genomes in monocytes fuel T cell activation, vascular and neuronal injury despite loss of replication-competent genomes, highlighting the importance of defective genomes as immune drivers.

Cluster 4 (low reservoir) demonstrated an increase in late CD8 T cell activation (low CD69^+^, high HLA-DR^+^/CD38^+^ and HLA-DR^+^/CD38^–^), CD4 and CD8 effector memory, and iNCMs, with a unique recovery of NK cell phenotype ratios. Interestingly, despite having the lowest reservoir burden, this cluster exhibited the most robust immune analyte signature with elevated levels of immune analytes associated with inflammation (IFN-γ, IP-10), trafficking (MIG, I-TAC, MIP-3β), tissue repair (SDF-1α), lymphocyte survival and proliferation (BDNF), and myeloid recruitment/development (MCP-1, M-CSF). This unique signature was surprising and suggests that increases in specific combinations of immune markers may be beneficial, resulting in a lower monocyte reservoir. Specifically, elevations in BDNF are associated with improved neuronal health, T cell function and proliferation (66), and NK cell maturation and function (67). SDF-1α, also known as CXCL12, can inhibit viral entry by blocking CXCR4 (68) and has been used therapeutically to promote tissue repair and reduce inflammation in chronic inflammatory conditions (69, 70). These analytes, in additional to chemokines associated with CD8 and NK function and trafficking, likely contribute to the CD8 activation and NK recovery signature observed and suggest 2 possible outcomes: (a) they promote an immune landscape that is unfavorable for the maintenance of the monocyte reservoir, or (b) despite a low proviral burden, immune activation persists compared with PWoH.

Cluster 5 (elevated intact) was strongly associated with altered monocyte populations, increased MCP-1, and decreased sVEGFR-1. The decreased proportions of monocytes, specifically CCR2^+^ classical and intermediate subsets, suggest that there are fewer monocytes capable of responding to MCP-1 in the blood and trafficking to the tissues. These data suggest 2 hypotheses; the first is that the specific decrease in monocytes may reflect poorer cell health when high reservoirs are present in this cell type as has been previously reported for CD4 T cells (15–17). The second is that CCR2^+^ monocytes respond to elevated concentrations of MCP-1 and egress from the blood to enter tissues. Given our current study design, we cannot determine which hypothesis is correct. However, there is substantial literature that supports that MCP-1 drives CCR2^+^ monocyte migration across endothelial barriers, such as the blood brain barrier (BBB), resulting in increased neuroinflammation (71, 72). Consistent with this interpretation, intact monocyte genomes have been linked with poorer cognitive performance in virally suppressed PWH, suggesting monocyte reservoirs may contribute to persistent neuroinflammation and neuronal injury despite ART (11). Decreased sVEGFR-1, an analyte that is secreted by endothelial cells and monocytes and acts as a soluble decoy receptor to neutralize VEGF and PlGF (73), suggests reduced vascular factor sequestration resulting in promotion of angiogenesis, vascular permeability, and inflammatory activation that can contribute to disease progression at endothelial barriers further supporting this hypothesis (74). Together, these observations suggest monocyte reservoirs may be linked to vascular dysregulation and neuroinflammation and contribute to immune heterogeneity during suppressive therapy compared with PWoH.

Several limitations should be considered when interpreting our findings. First, the cross-sectional design of this study limits our ability to infer causal relationships between reservoir burden and immune landscapes. Longitudinal studies are needed to determine whether specific reservoir profiles predict inflammatory profiles. Second, due to limited cell availability, we were only able to assess HIV genomes in total monocyte populations rather than perform subset-specific IPDA measurements, thus impairing our ability to precisely localize the reservoir within monocytes. Given the exploratory, high-dimensional nature of the immune landscape analysis, *P* values were corrected using an exploratory FDR threshold of 15%, an approach commonly used to prioritize biologically coherent signal patterns while limiting false positives. Results are therefore interpreted in a hypothesis-generating framework to inform future mechanistic and longitudinal studies. Additionally, as we relied on previously generated immunophenotyping and soluble analyte data, we did not have immune data available for all participants included in the reservoir clustering analysis, which may have reduced our statistical power. Finally, given these studies were designed to examine neurological specific comorbidities, we did not have data on other relevant factors (e.g., CVD, diabetes), nadir CD4 count, or timing of ART initiation. Available comorbidity data were focused on lifetime mental health and substance use. While expected differences were observed between PWH and PWoH, no differences were observed across reservoir phenotype clusters.

Collectively, our findings demonstrate that there are distinct reservoir phenotypes in virally suppressed PWH, and the heterogeneity in these phenotypes are associated with unique immune landscapes compared with PWoH. By integrating monocyte and CD4 T cell reservoirs, this study reveals that genomic integrity and cell type specificity shape reservoir phenotypes during ART. The presence of monocyte-centric phenotypes suggests that monocyte reservoirs may play a role in influencing immune activation despite viral suppression, though additional studies are needed to determine if the functionality of monocytes is altered based on reservoir phenotype as this study was primarily descriptive in nature. Incorporating reservoir phenotyping into clinical monitoring may help stratify individuals into specific inflammatory profiles that can be therapeutically targeted to reduce inflammation to the levels observed in PWoH leading to immune recovery. Additionally, the identification of well-defined clusters of PWH will allow for the development precision therapeutic approaches that are effective specific populations rather than broadly applied to all PWH. Further research into understanding how reservoir composition influences immune activation and function will be crucial for achieving stable immunologic recovery and future treatment strategies for HIV-associated comorbidities.

## Methods

### Sex as a biological variable.

Sex was not evaluated as a biological variable. Virally suppressed men (40%) and women (60%) with HIV were equally included in this study.

### Participants.

For the present analyses, banked PBMCs and previously acquired immune data (flow cytometry and soluble analytes) from 2 Johns Hopkins Brain Health Program (JH-BHP) studies conducted in Baltimore, Maryland, USA. The combined dataset included 164 PWH and 63 PWoH, with corresponding demographic, clinical, and behavioral data available for all participants. Inclusion criteria included age 18 to 90, English-speaking, ability to provide informed consent and travel to the study site, and for PWH self-reported consistent ART use. Exclusion criteria included current untreated hypertension or diabetes, head injury within the past year resulting in loss of consciousness for more than 1 hour, positive urine toxicology screen or breathalyzer (excluding cannabis), history of AIDS-defining or other neurologic disorder, history of Axis I psychosis based on the Structured Clinical Interview for DSM (SCID)-5, evidence of acute intoxication or withdrawal, or substance use disorder within the past 6 months. Lifetime mental health disorders and substance use histories were permitted and assessed as part of the parent studies. No new samples were collected, or assays performed for this analysis.

### IPDA.

To evaluate the composition of the HIV reservoir, we used the IPDA to quantify intact, defective, including both 3′ deleted/hypermutated and 5′ deleted/hypermutated, and total HIV genomes in monocytes and CD4^+^ T cells (10, 11, 14). In brief, TLR2^+^ monocytes were isolated from frozen participant PBMCs using a biotinylated TLR2 antibody (clone TL2.1, Invitrogen) and antibiotin magnetic beads (Miltenyi Biotec). CD4 T cells were then isolated from TLR2- flow through using a negative CD4 selection kit (Miltenyi Biotec). TLR2 selection cell purity was verified by flow cytometry with a panel including TLR2 (clone 11G7, BD Biosciences), CD3 (clone SP34-2, BD Biosciences), and CD4 (clone L200, BD Biosciences and Live/Dead Near IR stain (Invitrogen). Following isolation, cells were lysed in AllPrep buffer of RLT plus+βME and DNA was extracted using AllPrep DNA/RNA kits (Qiagen). The primers, probes, and reaction conditions have been previously reported (10). Each sample was assessed across multiple replicates (3–6 replicates) to ensure data reliability, aiming for a minimum of one million input cells per sample or maximum available as quantified based on the cellular gene RPP30. In cases where no signal was detected, despite sufficient cell numbers, the samples were assigned a value of ‘0.1’ as the LOD. All monocyte IPDA results were adjusted to account for potential CD4^+^ T cell contamination as shown in Supplemental Table 5 and previously described (10, 11). In brief, we used corresponding IPDA results from CD4^+^ T cells and flow cytometry to estimate the number of CD4^+^ T cells present per million monocytes and calculate the potential contribution of intact, 3′ defective, or 5′ defective signals from those contaminating cells. These estimated values were then subtracted from the monocyte IPDA measurements to ensure more accurate quantification of the monocyte-specific reservoir. In addition, all results were adjusted for DNA shearing as previously described (14).

### Immunophenotyping.

Immunophenotyping data available for analysis consisted of basic and in-depth phenotyping panels and were acquired on whole-blood samples that were stained within 2 hours of collection as previously described (33, 39). The basic phenotyping panel was used to determine the prevalence of CD4 and CD8 T cells, CD4/CD8 ratio and monocyte subsets (classical, intermediate, nonclassical) and consisted of: CD3-V500 (clone SP34-2 BD, Biosciences), CD4-PerCP-Cy5.5 (clone L200, BD Biosciences), CD8a-BV570 (clone RPA-T8, BioLegend), TLR2-AF488 (clone 11G7 BD, Biosciences), CD14-BV650 (clone M5E2 BD, Biosciences), and CD16-AF700 (clone 3G8, BioLegend). The in-depth phenotyping panel was used to determine the prevalence of CD4 and CD8 T cell activation and memory phenotypes, monocyte activation and trafficking phenotypes, NK cells and B cells and included: CD3-BV510 (clone OKT3, BioLegend), CD4-BV421 (clone RPA-T4, BioLegend), CD8a-BV570/APC-Cy7 (clone RPA-T8, BioLegend), TLR2-FITC (clone 11G7, Invitrogen), CD14-BV650 (clone M5E2, BioLegend), CD16-BV570 (clone 3G8, BioLegend), CD69-APC/Fire 750 (clone FN50, BioLegend), HLA-DR-BV650 (clone G46-6/L243, BD Biosciences), CD38-PerCP-Cy5.5 (clone HIT2, BioLegend), CD11a-PE-Cy7 (clone HI111, BioLegend), CD95-PE (clone DX2, BioLegend), CD28-APC-R700 (clone CD28.2, BD Biosciences), CD62L-APC (clone DREG-56, BioLegend), CD45RO-FITC (clone UCHL1, BioLegend), CD42a-R718 (clone ALMA.16, BD Biosciences), CCR2-APC (clone 48607, R&D Systems), ALCAM-BV421 (clone 3A6, BD Biosciences), CD56-APC (clone 5.1H11, BioLegend), CD159a-PE (clone S19004C, BioLegend), and CD20-R718 (clone 2H7, BD Biosciences). TLR2 specificity as a monocyte marker was previously confirmed (39). The gating strategy for the basic panel has been previously described (33). The gating strategy for the in-depth panel is presented in Supplemental Figure 9. All gating strategies were determined using fluorescence minus 1 controls. Voltage settings were standardized to daily CS&T Research Beads (BD Biosciences) using application settings determined based on fluorescent intensities in FACSDiva. Data were acquired on a BD LSRFortessa (BD Biosciences) within 2 hours of staining and analyzed using FlowJo (version 10.9.0; BD Biosciences). A complete blood count (CBC) with differential was also acquired and used to calculate absolute cell counts.

### Soluble immune markers.

Soluble immune data available for analysis consisted of specific markers selected to assess general inflammation (IFN-γ, IL-6, C-reactive protein [CRP], CD163, CD14, IL-18, IFN-γ Inducible Protein [IP]-10), neuroinflammation (TNF-related apoptosis-inducing ligand [TRAIL], IL-10, brain-derived neurotrophic factor [BDNF], soluble CD40 ligand [sCD40-L], Neurofilament Light Chain [NF-L], vWF), leukocyte recruitment (macrophage inflammatory protein [MIP]-1α, MIP-1β, MIP-3 β, stromal cell-derived factor [SDF]-1α, monocyte chemoattractant protein [MCP]-1, macrophage colony-stimulating factor [M-CSF], IFN-inducible T-cell α chemoattractant [I-TAC], monokine Induced by IFN-γ [MIG], serum amyloid A [SAA]), vascular function (Clusterin, Cystatin C, Tie-2, placental growth factor [P1GF], vascular endothelial growth factor [VEGF]-A, VEGF receptor [R]-1), and endothelial integrity (vascular cell adhesion molecule [VCAM]-1, intercellular adhesion molecule [ICAM]-1, matrix metalloproteinase [MMP]-2, MMP-9, Fractalkine). Plasma analytes were assessed using Meso Scale Discovery U-PLEX and R-PLEX immunoassays according to the manufacture’s recommendation. Plasma was aliquoted and stored at –80°C, with no freeze-thaw cycles prior to analysis to preserve sample integrity. Analyte concentrations were calculated using the Discovery Workbench software (Meso Scale Discovery) and log transformed prior to further analysis.

### Immune data analysis.

Immune variables (basic, in-depth, and soluble) from PWoH were standardized by converting each to a *z* score, which was calculated by subtracting the sample mean and dividing by the standard deviation across all values resulting in an interpretable scale with a mean of 0 and a standard deviation of 1. However, raw values are provided in Supplemental Table 4. The mean values from PWoH were then used to calculate a normalized *z* score for PWH by subtracting the PWoH mean from the PWH value and dividing by the PWoH standard deviation for all values. Normalized *z* score values outside of the 5% and 95% intervals were removed from analysis (Supplemental Table 6). This normalization results in the data being represented as a difference compared with PWoH and reported for each reservoir cluster as an average normalized *z* score. Range of normalized *z* scores for each immune measure in PWoH and reservoir cluster are displayed in Supplemental Figures 5 and 7.

### Sensitivity analysis.

To assess the robustness of clustering to the handling of values below the LOD we performed a sensitivity analysis comparing our primary GMM solution which assigned a value of 0.1 to undetectable IPDA data points to models assigning alternative values (0.05, 0.001, and 0). Cluster agreement was quantified using the ARI, calculated via the mclust package (version 6.1.2) in R. The ARI evaluates the similarity between 2 clustering solutions by examining all possible pairs of participants and determining the consistency of their grouping while adjusting for the level of agreement expected by chance. An ARI of 1.0 indicates perfect agreement, whereas a score of 0 indicates agreement no better than random assignment (75). ARI values exceeded 0.9 across all comparisons, indicating near-identical participant assignments. Visual inspection using alluvial plots with the ggforce package (version 0.5.0) demonstrated minimal migration between clusters, supporting that cluster structure is driven by underlying biological variation rather than imputation of detectable values.

Following unsupervised clustering, Wilcoxon matched-pairs signed-rank tests were conducted to compare IPDA measures (intact, 3′ del, 5′ del and total) between paired monocytes and CD4 T cells. Kruskal-Wallis 1-way ANOVA was conducted to compare the means of each IPDA measure across reservoir clusters, with a correction for multiple comparisons using Dunn’s test. Multiple Mann-Whitney *U* tests were conducted to compare each measure within reservoir clusters across cell types, with a correction for multiple comparisons using a 2-stage step-up False Discovery Rate (FDR) of 5%. Pearson correlations analyses were used to determine if CD4 T cells contributed to monocyte signal. For exploratory immune analysis, multiple Mann-Whitney *U* tests were conducted to compare PWH normalized *z* scores to the PWoH *z* scores in each reservoir cluster, with a correction for multiple comparisons using a 2-stage step-up exploratory False FDR of 15%. The immune-phenotype data were treated as one batch of variables with 41 unique variables. The soluble immune analyte data were treated as separate batch of variables with 33 unique variables. Chi square tests were used to determine differences in demographic data between groups and clusters. Data analysis was performed in GraphPad Prism (version 10.6.1) and differences with *P*-values less than 0.05 were considered statistically significant.

### Statistics.

To determine clusters of participants with distinct cell-type reservoir patterns we used a multi-step approach integrating dimension reduction through PCA with varimax rotation (*SciDataReportR* package version 12.8.0) (76) and Gaussian Mixture Model (GMM)-based clustering (77) in R, version 4.5.0, using the *tidyLPA* package (78), which provides a unified interface for estimating and comparing latent profile models across multiple parameters and cluster numbers. GMM clustering was selected over distance-based methods because it supports flexible cluster shapes and provides probabilistic membership estimates. Prior to computing the principal components, 6 reservoir variables (intact, 3′ defective, 5′ defective copies per million cells) from both CD4 T cells and monocytes were first log transformed and standardized to *z* scores by subtracting the sample mean and dividing by the standard deviation across all participants for each variable. The *z* scores allow the variables to be scaled and compared on the same metric, improving comparability and enhancing the interpretability and consistency of the results. By transforming the original correlated variables into a set of uncorrelated components, PCA helps address multicollinearity, which can obscure independent associations between the original variables and the outcomes of interest. The retained PCA components were selected based on their ability to sufficiently explain 80% of the variance within the dataset. GMM clustering, an unsupervised clustering technique that uses probabilistic modeling to identify natural groupings, was applied to the retained principal components to identify clusters of participants based on their HIV reservoir profile. This allows for the categorization of distinct reservoir phenotypes which can then be used in subsequent analyses to explore their differential interactions with immune measures. When compared with traditional k-means clustering, GMM clustering supports the identification of complex and nuanced patterns, allowing for more flexible cluster shapes (79). The optimal number of clusters was determined using several statistical fit indices, including the Bayesian Information Criterion (BIC), Akaike Information Criterion (AIC), Entropy, and Log-Likelihood (LogLik). We evaluated solutions ranging from 2 to 10 clusters across 3 distinct model structure: Model 1 (equal variance, zero covariance), Model 2 (varying variance, zero covariance), and Model 3 (equal variance, equal covariance). The final model selection was based on the configuration that minimized AIC and BIC and maximized LogLik and Entropy, ensuring robust cluster separation and optimal fit for the reservoir data. Finally, a 0.75 posterior membership probability threshold was applied to exclude participants whose profiles were poor fits for their assigned clusters. This threshold ensures that the final clusters represent distinct and reliable reservoir phenotypes. By employing this combination of PCA for dimensionality reduction and GMM clustering for pattern identification, we established fine-tuned classification groups that can be leveraged in subsequent analyses to explore differential interactions between reservoir phenotypes and immune measures.

### Study approval.

The present study utilized bank samples and previously acquired data from 2 JH-BHP studies conducted in Baltimore, Maryland, USA. All participants provided written informed consent under protocols approved by the Johns Hopkins University IRB, and both studies were conducted in accordance with the principles of the Declaration of Helsinki.

### Data availability.

All values for all data presented in graphical form are provided in the Supporting data values file. The coding pipeline to reproduce the clustering is available at: https://github.com/BHPDataSci/HIVReservoirPhenotyping (CommitID: a0c1c492e391278f3ce271edbcc902c8cea7aad2).

## Author contributions

RTV and LR designed the study. RW, LP, HSR, and KH acquired samples and performed experiments. RW, LP, AB, ENS, HSR, and RD performed analyses. RW and RTV wrote the manuscript. RW, RTV, LR, and RD revised manuscript. RTV, JC, and LR funded the study. All authors read and approved the final manuscript.

## Conflict of interest

The authors have declared that no conflict of interest exists.

## Funding support

This work is the result of NIH funding, in whole or in part, and is subject to the NIH Public Access Policy. Through acceptance of this federal funding, the NIH has been given a right to make the work publicly available in PubMed Central.

- R01 MH127981 (Veenhuis)
- R01 MH113512 (Rubin)
- R01 MH113512-S (Rubin)
- R01 MH125300 (Coughlin, Rubin)
- P30 MH075673 (Rubin, Slusher)
- Johns Hopkins Institute for Clinical and Translational Research (ICTR, UM1 TR004926).

## Supplementary Material

Supplemental data

ICMJE disclosure forms

Supplemental table 2

Supplemental table 3

Supplemental table 4

Supporting data values

## Figures and Tables

**Table 1:**
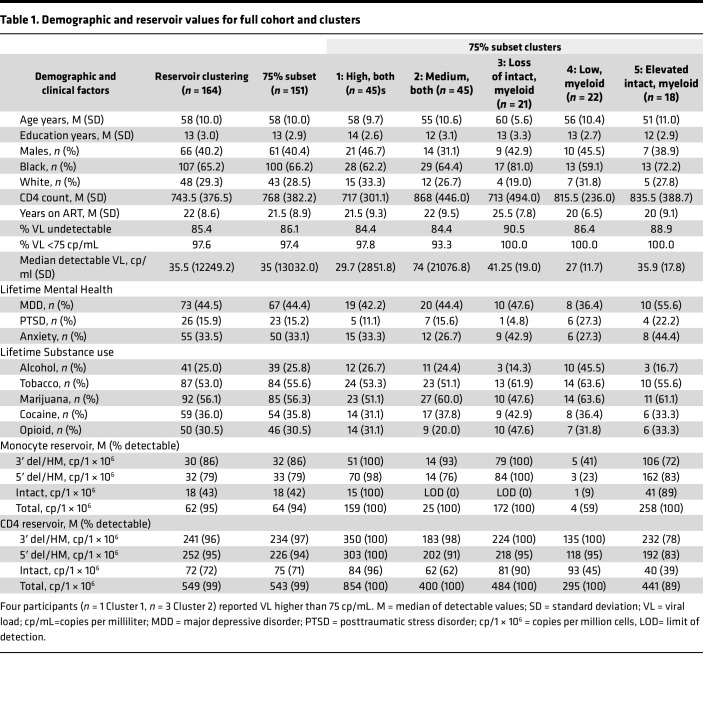
Demographic and reservoir values for full cohort and clusters

**Table 2:**
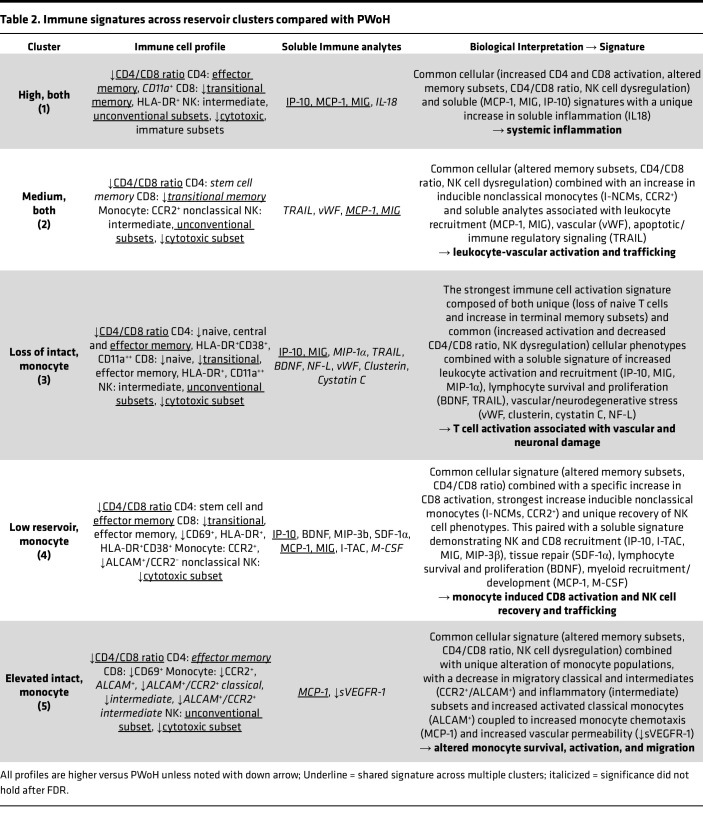
Immune signatures across reservoir clusters compared with PWoH

## Version 1 05/28/2026

In-Press Preview

## Version 2 07/22/2026

Electronic publication
