# Supplementary material for: Identification of distinct HIV reservoir phenotypes and associated immune landscapes
Source: JCI Insight. 2026 May 28;11(14):e203765. doi: 10.1172/jci.insight.203765 (PMC13461171; doi:10.1172/jci.insight.203765)
Supplement: Supplementary file 1 [file jciinsight-11-203765-s312.pdf]

Supplemental Table 1. Demographics and sample size for immune analyses

Demographic and clinical factors	Basic phenotyping[A]		In-depth phenotyping[B]		Immune analyte measures[C]	
	PWoH (n=63)	vsPWH* (n=144)	PWoH (n=63)	vsPWH (n=92)	PWoH (n=49)	vsPWH (n=86)
age years, M (SD)	63 (13.4)	58 (9.7)	63 (13.4)	58 (10.7)	60.5 (22.0)	58.5 (10.1)
education years, M (SD)	14 (3.8)	13 (2.9)	14 (3.8)	14 (3.1)	14 (3.5)	14 (3.2)
males, n (%)	41 (63.1)	59 (41.0)	41 (63.1)	59 (64.1)	34 (70.8)	56 (65.9)
Black, n (%)	28 (43.1)	95 (66.0)	28 (43.1)	52 (56.5)	22 (45.8)	48 (56.5)
White, n (%)	31 (47.7)	41 (28.5)	31 (47.7)	34 (37.0)	24 (50.0)	33 (38.8)
**Lifetime Mental Health**						
lifetime MDD, n (%)	11 (16.9)	63 (43.8)	11 (16.9)	35 (38.0)	6 (12.2)	32 (37.2)
lifetime PTSD, n (%)	9 (13.8)	23 (16.0)	9 (13.8)	19 (20.7)	4 (8.2)	16 (18.6)
lifetime Anxiety, n (%)	20 (30.8)	47 (32.6)	20 (30.8)	38 (41.3)	12 (24.5)	35 (40.7)
**Lifetime Substance use**						
alcohol, n (%)	12 (18.5)	37 (25.7)	12 (18.5)	24 (26.1)	12 (24.5)	22 (25.6)
tobacco, n (%)	20 (30.8)	81 (56.3)	20 (30.8)	48 (52.2)	17 (34.7)	47 (54.7)
marijuana, n (%)	16 (24.6)	80 (55.6)	16 (24.6)	48 (52.2)	14 (28.6)	43 (50.0)
cocaine, n (%)	14 (21.5)	51 (35.4)	14 (21.5)	27 (29.3)	11 (22.4)	27 (31.4)
opioid, n (%)	9 (13.8)	44 (30.6)	9 (13.8)	25 (27.2)	6 (12.2)	24 (27.9)
**Reservoir cluster participants with immune data, n (%)**						
1: High, both (n=45)		42 (93.3)		29 (64.4)		29 (64.4)
2: Medium, both (n=45)		42 (93.3)		22 (48.9)		19 (42.2)
3: Loss of intact, myeloid (n=21)		21 (100.0)		16 (76.2)		16 (76.2)
4: Low, myeloid (n=22)		22 (100.0)		14 (63.6)		12 (54.5)
5: Elevated intact, myeloid (n=18)		17 (94.4)		11 (61.1)		10 (55.6)

*Note: 4 participants with viral load higher than 75cp/mL were omitted from basic phenotyping analysis and did not have data in other immune areas.
PWoH=people without HIV; vsPWH=virally suppressed people with HIV; M=median; SD=standard deviation, MDD=major depressive disorder; PTSD=post traumatic stress disorder
A cell types: CD4 and CD8 T cells, CD4:CD8 ratio and monocyte subsets (classical, intermediate, non-classical)
B cell types: CD4 and CD8 T cell activation and memory phenotypes, monocyte activation and trafficking phenotypes, NK cells and B cells
C panels include general inflammation, neuroinflammation, leukocyte recruitment, vascular function, and endothelial integrity

Supp tables 2-4 are provided in excel form

Supplemental table 5. Example calculations for CD4 signal removal from monocyte IPDA

	Variable	Example value
	%CD4+ cells post selection	2%
	Intact signal per 1e6 CD4	100 copies/1e6 CD4
	Intact signal per 1e6 monocytes	10 copies/1e6 monocytes
	**Calculation steps**	**Example**
1	**estimate of CD4# per 1e6 monocytes** = %CD4+ post selection cells * 1e6 cells	0.02*1,000,000 = **20,000 CD4s**
2	**intact signal per CD4** = intact signal per 1e6 CD4 / 1e6 cells	100/1,000,000= **0.0002 copies/CD4**
3	**estimate of intact signal from CD4** = estimate of CD4# per million monocytes * intact signal per CD4	20,000 CD4 * 0.0002 cp/CD4= **4 copies**
4	**CD4 adjusted intact per 1e6 monocytes** = DSI adjusted # of intact per 1e6 monocytes – estimate of intact signal from CD4	10 – 4 = **6 copies/1e6 monocytes reported**

*the same calculations are completed for 3’ del/HM and 5’del/HM values. Total provirus is the sum of the CD4 adjusted values.

Supplemental Table 6. Normalized z-score outlier removal

Immune markers	% removed
**Bulk T cells**	
CD3+ T cells % CD4+	1.87
CD3+ T cells % CD8+	1.87
CD3+ T cells CD4/CD8 ratio	1.87
**CD4 T cell memory and activation**	
CD4 T cells % effector memory	1.87
CD4 % HLA-DR+ CD38-	1.40
**CD8 T cell memory and activation**	
CD8 T cells % effector memory	0.93
CD8 % T stem cell memory	0.47
CD8 % HLA-DR+ CD38-	0.93
CD4 % HLA-DR+ CD38+	0.93
**Monocytes**	
TLR2+ % classical monocytes	1.87
TLR2+ % inter. monocytes	1.87
TLR2+ % non-classical monocytes	1.87
classical monocytes % CCR2+	1.87
inter. monocytes % CCR2+	1.87
non-classical monocytes % CCR2+	1.87
**NK cells**	
NK cells CD56 bright CD16dim	0.47
NK cells CD56- CD16+	0.47
NK cells CD56 dim CD16-	1.40
NK cells CD56 bright CD16-	0.93
**Absolute cell counts**	
CD8 T cells/uL	2.80
NK cells/uL	0.47
**Soluble immune analytes**	
P1GF	2.99
CD14	2.24
Cystatin C	1.49
MIP-1α	0.75
MCP-1	0.75
CD163	0.75
MMP-2	0.75
MMP-9	0.75
Tie-2	0.75

* If a marker is not listed, then 0% of the sample required removal.

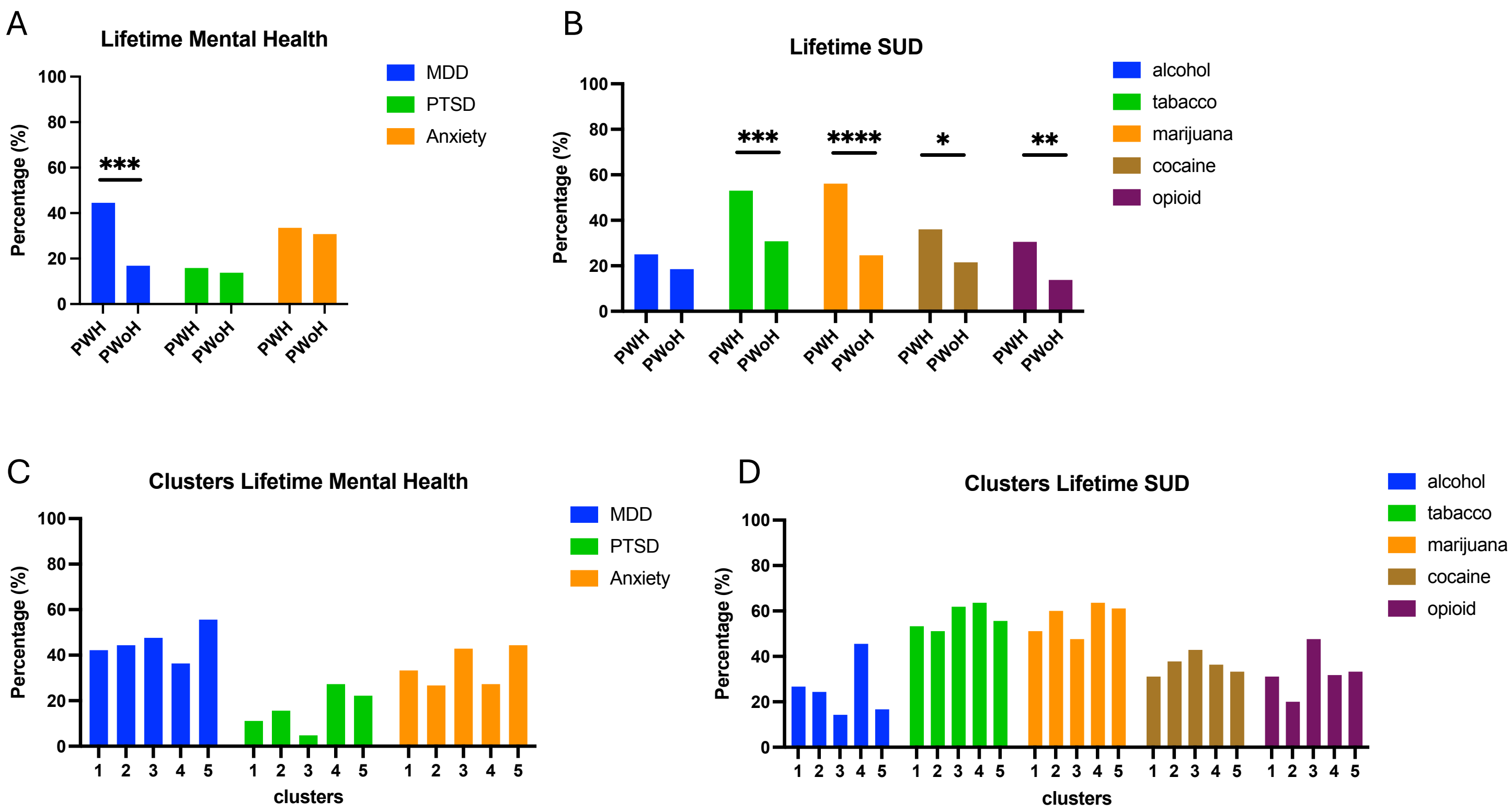

Supplemental Figure 1. Mental health and substance use disorder (SUD) prevalence in cohort and reservoir clusters. Percentage of people with HIV (PWH) and people without HIV (PWoH), reporting lifetime major depressive disorder (MDD), post traumatic stress disorder (PTSD) and anxiety (A). Percentage of PWH and PWoH reporting lifetime SUD (B). Percentage of PWH in each reservoir cluster reporting MDD, PTSD, and anxiety (C). Percentage of PWH in each reservoir cluster reporting SUD (D). Chi Sq test, *p<0.05, **p<0.01, ***p<0.001, ****p<0.0001.

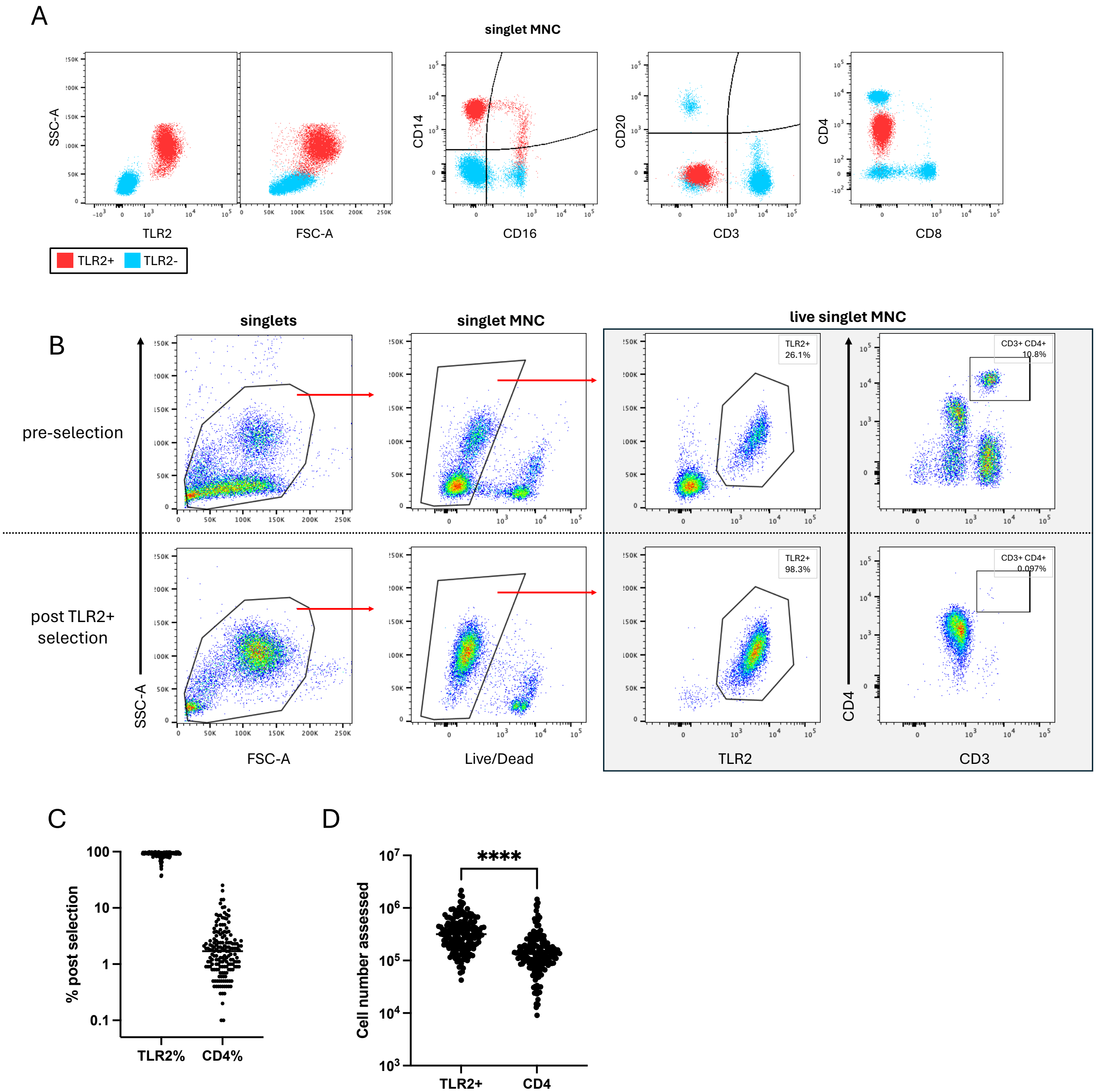

Supplemental Figure 2. TLR2 expression on monocyte and purity post selection. Representative whole blood phenotyping demonstrates that TLR2 uniquely captures all monocyte subsets in blood with minimal contamination by other cell types (A). Cells are first gated based on FSC-A vs. FSC-H followed by gating for mononuclear cells (MNC) to remove debris (not shown) . Singlet mononuclear cells (MNCs) are then gated based on their expression of TLR2+ (red) or TLR2- (blue) demonstrating that NK, B and T cells do not express TLR2. Representative flow cytometry data before and after TLR2 selection (B) of frozen PBMCs. Singlets are first gated based on FSC-A vs. FSC-H (not shown) followed by gating for MNCs to remove debris. The Live/Dead Near Infrared marker is then used to gate live cells. Live, singlet, MNCs are then gated based on their expression of TLR2 or on combined CD3+ CD4+ T cells.
Percent TLR2 purity and CD4 contamination post monocyte selection of 164 participants (C). Cell number assessed in monocyte and CD4 T cell IPDA, ****$p<0.0001$, Welch's T test (D).

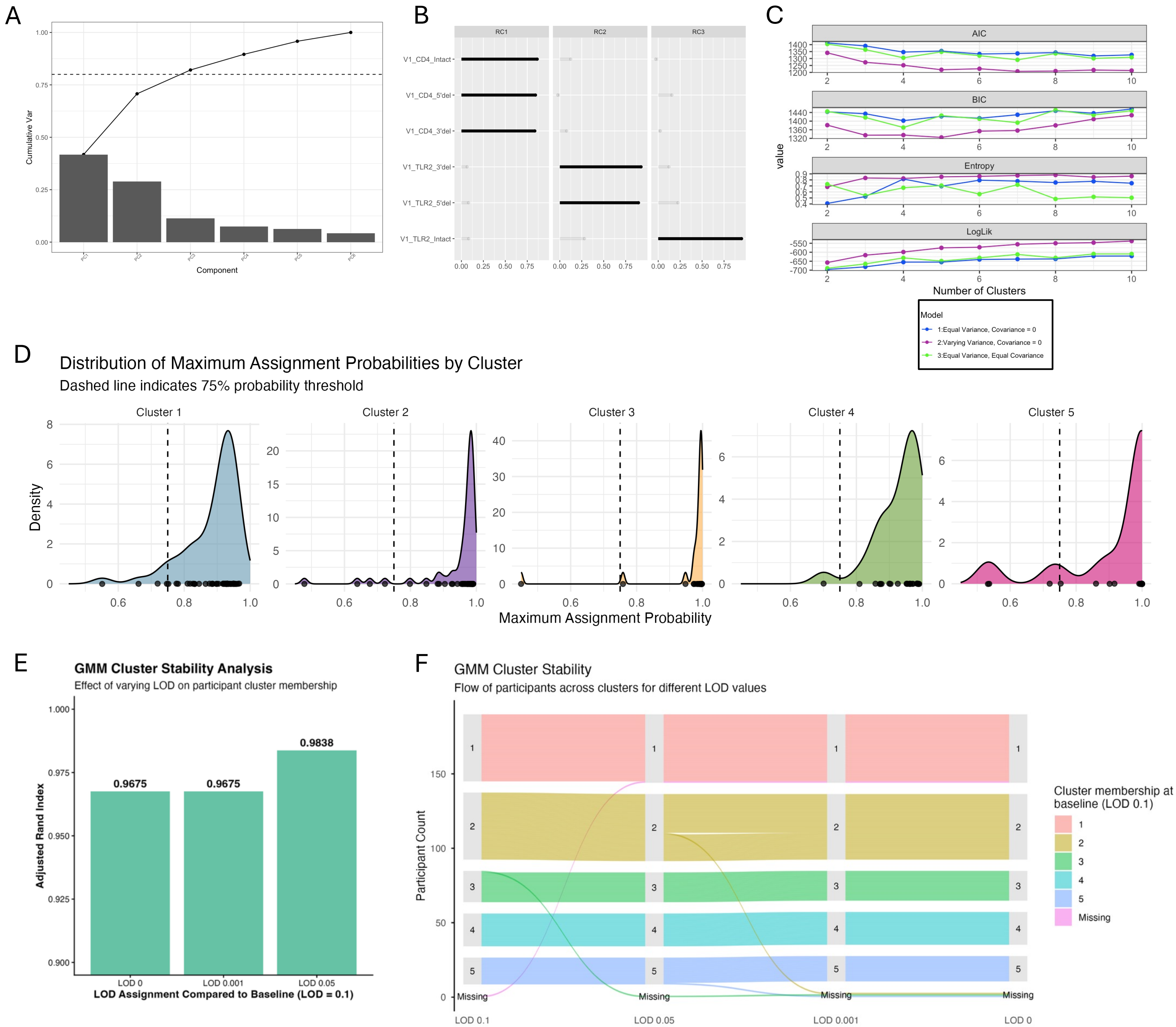

Supplemental Figure 3. Generation of the reservoir phenotype clusters. To determine clusters of participants with distinct cell-type reservoir patterns we used a multi-step approach integrating dimension reduction through principal components analysis and Gaussian Mixture Model-based (GMM) clustering. PCA component loading was determine based on greater that 75% of variability being accounted for (A) which results in 3 components (B). The optimal number of clusters was determined using the following statistical criteria: Bayesian Information Criterion (BIC) or Akaike Information Criterion (AIC), Entropy, and Log-Likelihood (LogLik). Each criterion was plotted across cluster numbers (from 2 to 10 clusters) for each model structure. Optimal clusters were selected based on the model that minimized AIC and BIC and maximized LogLik and Entropy for robust cluster assignments (C). 75% posterior membership probability threshold was applied to exclude participants whose profiles were poor fits for their assigned clusters. This threshold ensures that the final clusters represent distinct and reliable reservoir phenotypes (D). An Adjusted Rand Index (ARI) bar plot, which is a measure used to quantify the similarity between two clustering solutions by evaluating the consistency of participant cluster assignments (E). An alluvial plot to demonstrate individual participant movement across the different GMM solutions (F). The relative absence of crisscrossing lines serves as visual evidence that the cluster separation is driven by robust biological signals rather than artifacts of how undetectable values were handled.

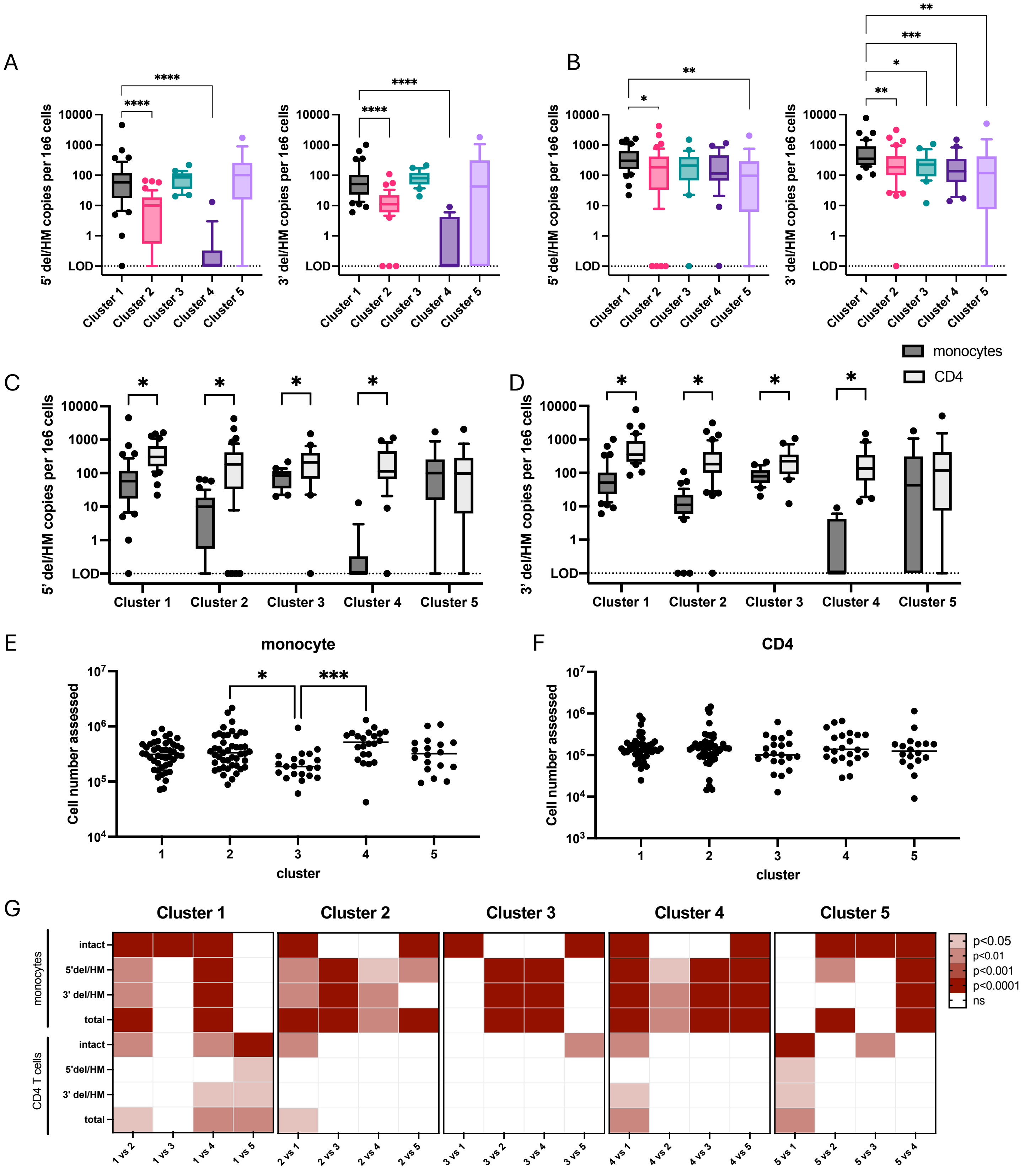

Supplemental Figure 4. 5' and 3' defective reservoir characteristics across clusters in monocytes and CD4 T cells. Comparison of absolute 5' defective and 3' defective genome values across clusters within monocytes (A) and CD4 T cells (B). Comparison of 5' defective (C) and 3' defective (D) genome copies between monocytes and CD4 T cells within clusters (C). Cell numbers assessed in IPDA per cluster for monocytes (E) and CD4 T cells (F). Additional statistical comparisons across all clusters, heat map displays significant p values (G). Sample size for cluster 1 n= 45, cluster 2 n=45, cluster 3 n=21, cluster 4 n=22, cluster 5 n=18, box and whiskers plots show 10-90 percentile, dots are data points outside of that range. Statistics were completed using either Kruskal-Wallis test (ANOVA for multiple groups A, B, E-G) with Dunn's correction for multiple comparisons or multiple Mann-Whitney T-tests (C-D) with a correction for multiple comparisons using a two-stage step-up False Discovery Rate (FDR) of 5%, *p<0.05, **p<0.01, ***p<0.001, ****p<0.0001. del/HM = deleted and hypermutated.

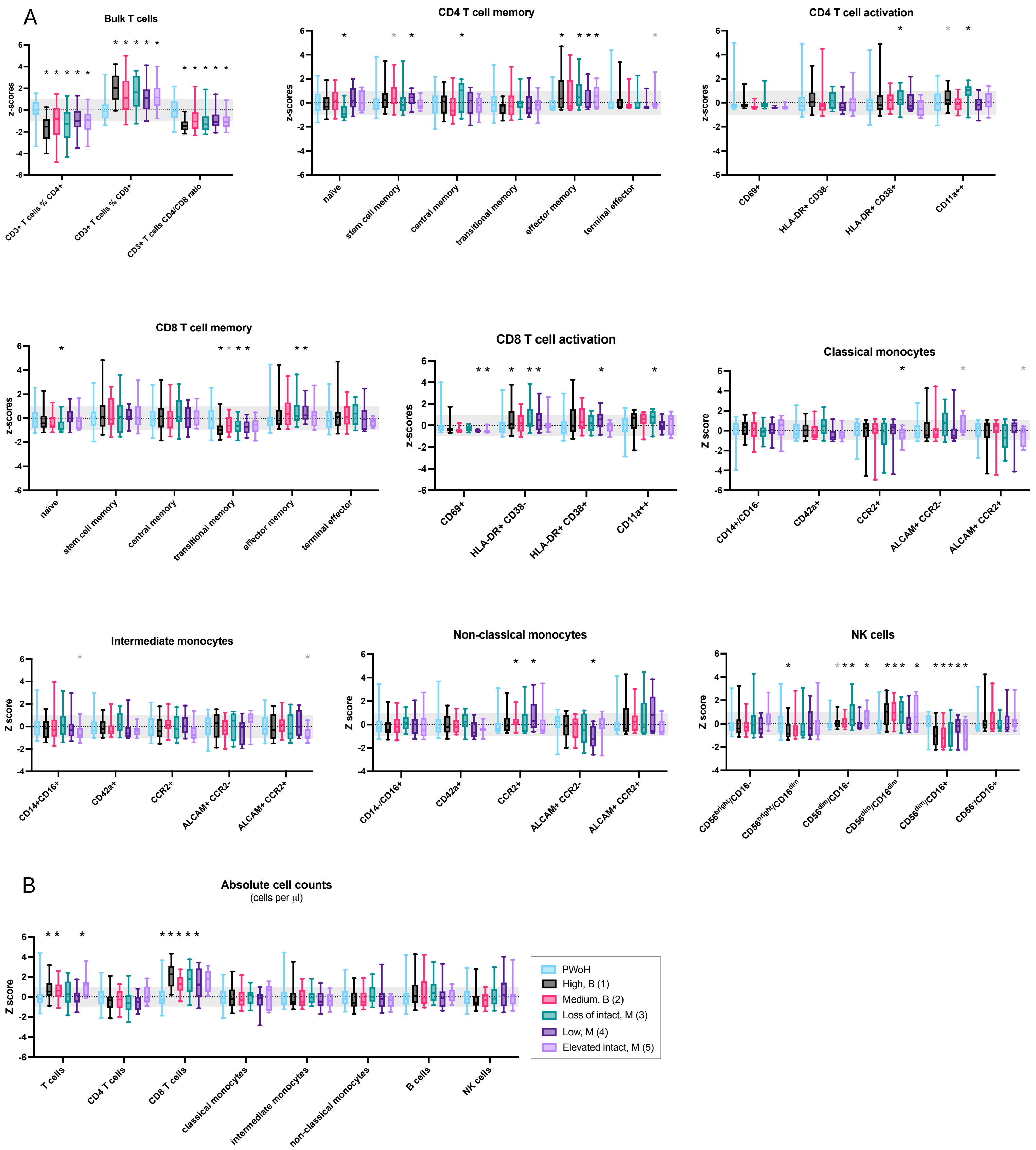

Supplemental Figure 5. Z scores of immune cell populations from PWoH and the 5 reservoir clusters. Z-score distributions of bulk T cells, CD4 memory and activation, CD8 memory and activation, monocytes subsets, and NK cell phenotypes (A). Z-score distributions of absolute cell counts (B). Statistics were completed using multiple Mann-Whitney T-tests between between PWoH and each reservoir cluster with a correction for multiple comparisons using a two-stage step-up exploratory False Discovery Rate (FDR) of 15%, asterisk indicates significance of $p<0.05$, specific p values are listed on corresponding heat map in figure 3. Black asterisk = significant post-FDR, gray asterisk = significant pre-FDR, but did not hold post correction .

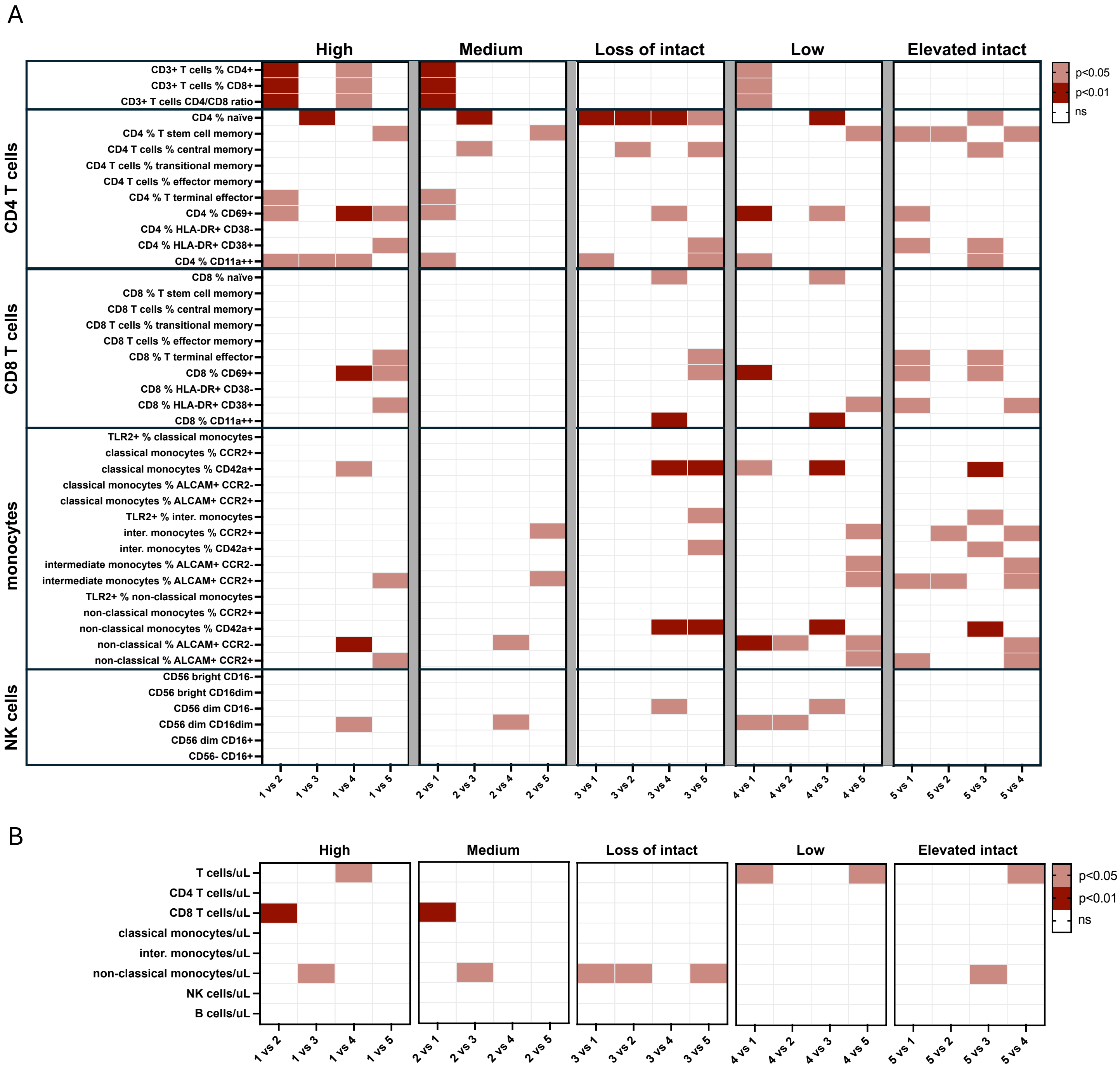

Supplemental Figure 6. Significant immune cell phenotype and absolute cell number comparisons between reservoir clusters. All significant immune phenotype (A) and absolute cell number (B) comparisons between cluster groupings are highlighted. White = not significant (ns), light red = $p<0.05$, dark red = $p<0.01$, comparisons are not FDR adjusted and intended to demonstrate that there are differences between clusters and as an exploratory analysis.

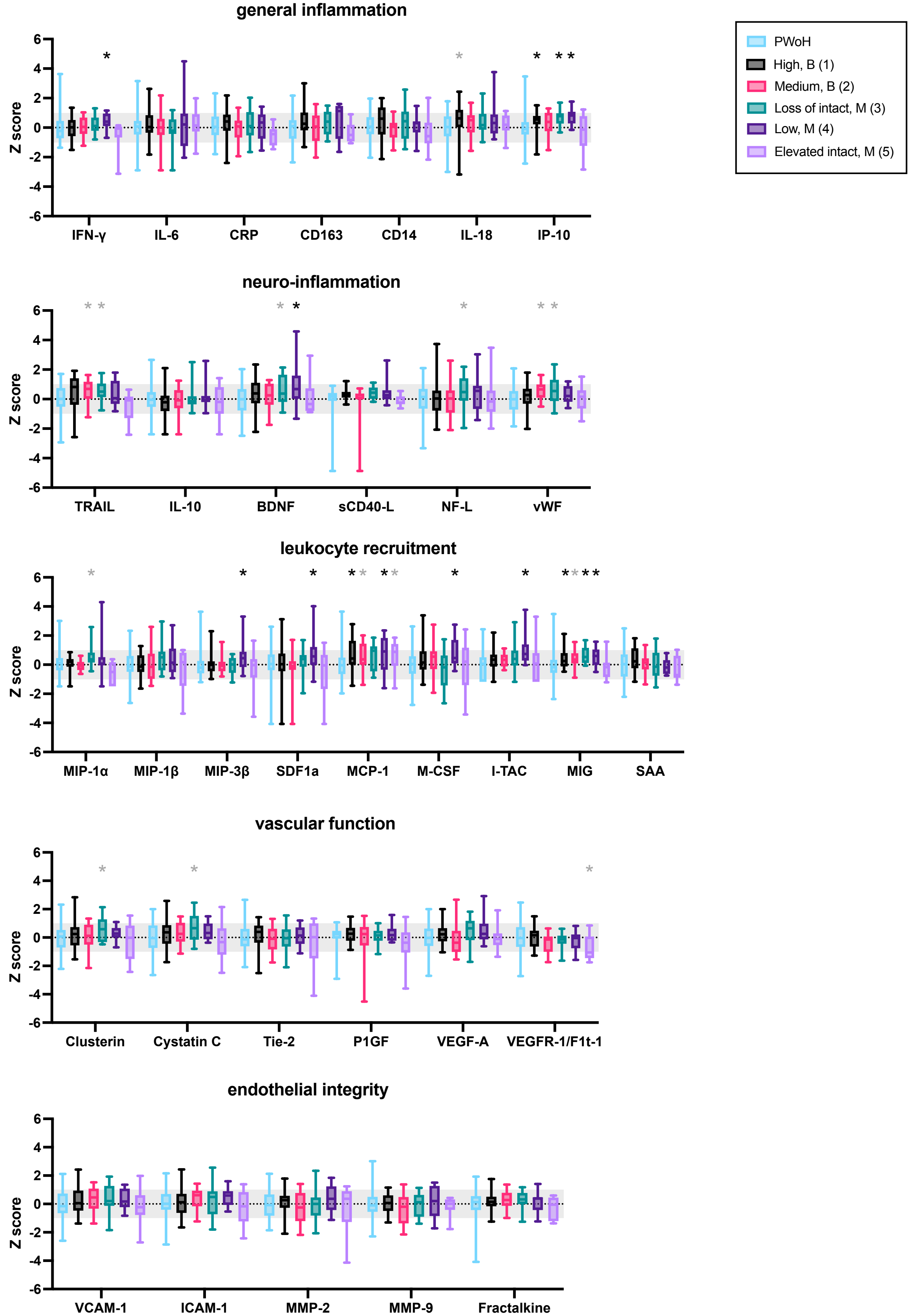

Supplemental Figure 7. Z scores of immune cell populations from PWoH and the 5 reservoir clusters. Z-score distributions of soluble analytes associated with general inflammation, neuro-inflammation, leukocyte recruitment, vascular function, and endothelial integrity. Statistics were completed using multiple Mann-Whitney T-tests between between PWoH and each reservoir cluster with a correction for multiple comparisons using a two-stage step-up exploratory False Discovery Rate (FDR) of 15%, asterisk indicates significance of p<0.05, specific p values are listed on corresponding heat map in figure 3. Black asterisk = significant post-FDR, gray asterisk = significant pre-FDR, but did not hold post correction.

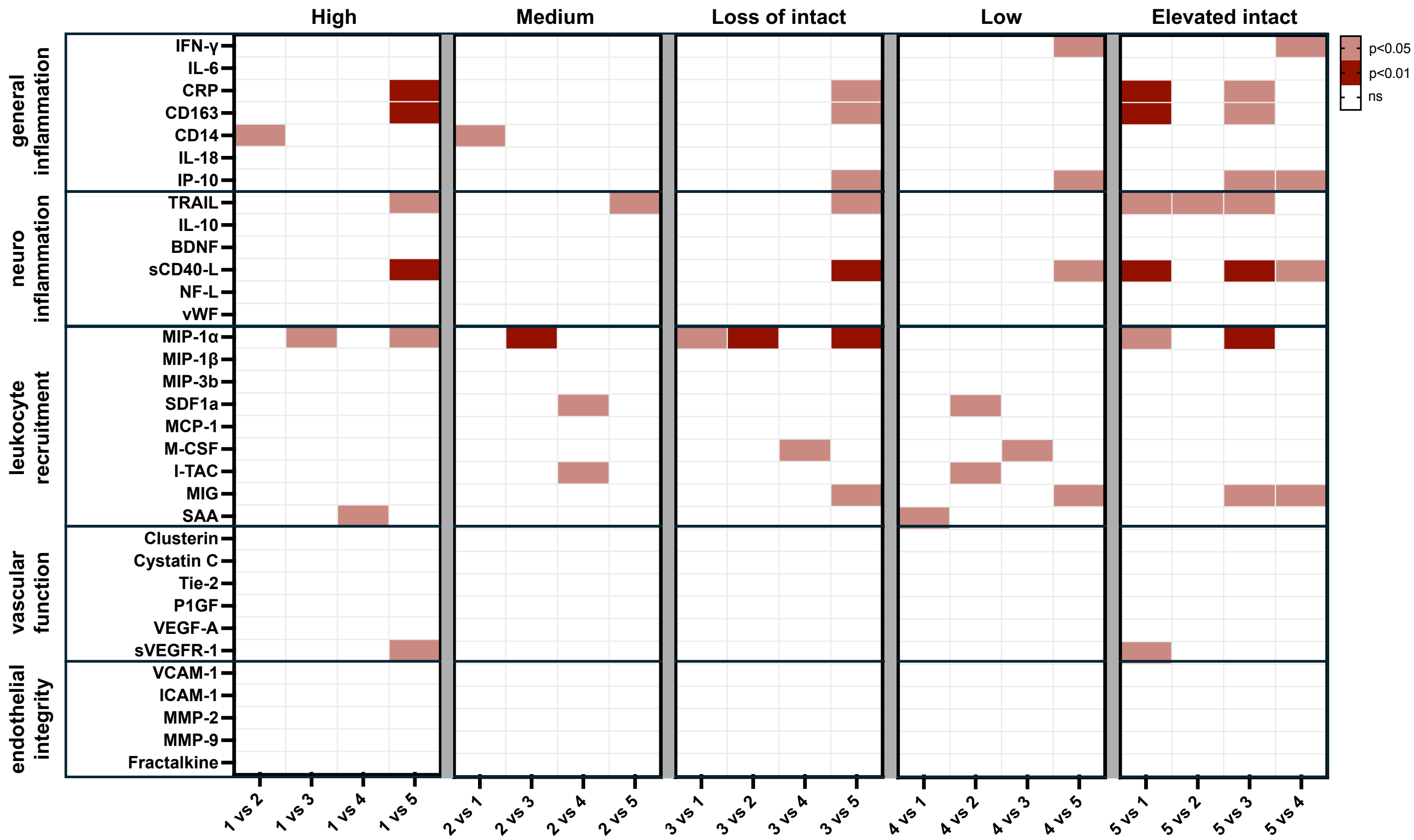

Supplemental Figure 8. Significant immune analyte comparisons between reservoir clusters. All significant immune analyte comparisons between cluster groupings are highlighted. White = not significant (ns), light red = $p<0.05$, dark red = $p<0.01$, comparisons are not FDR adjusted and intended to demonstrate that there are differences between clusters and as an exploratory analysis.

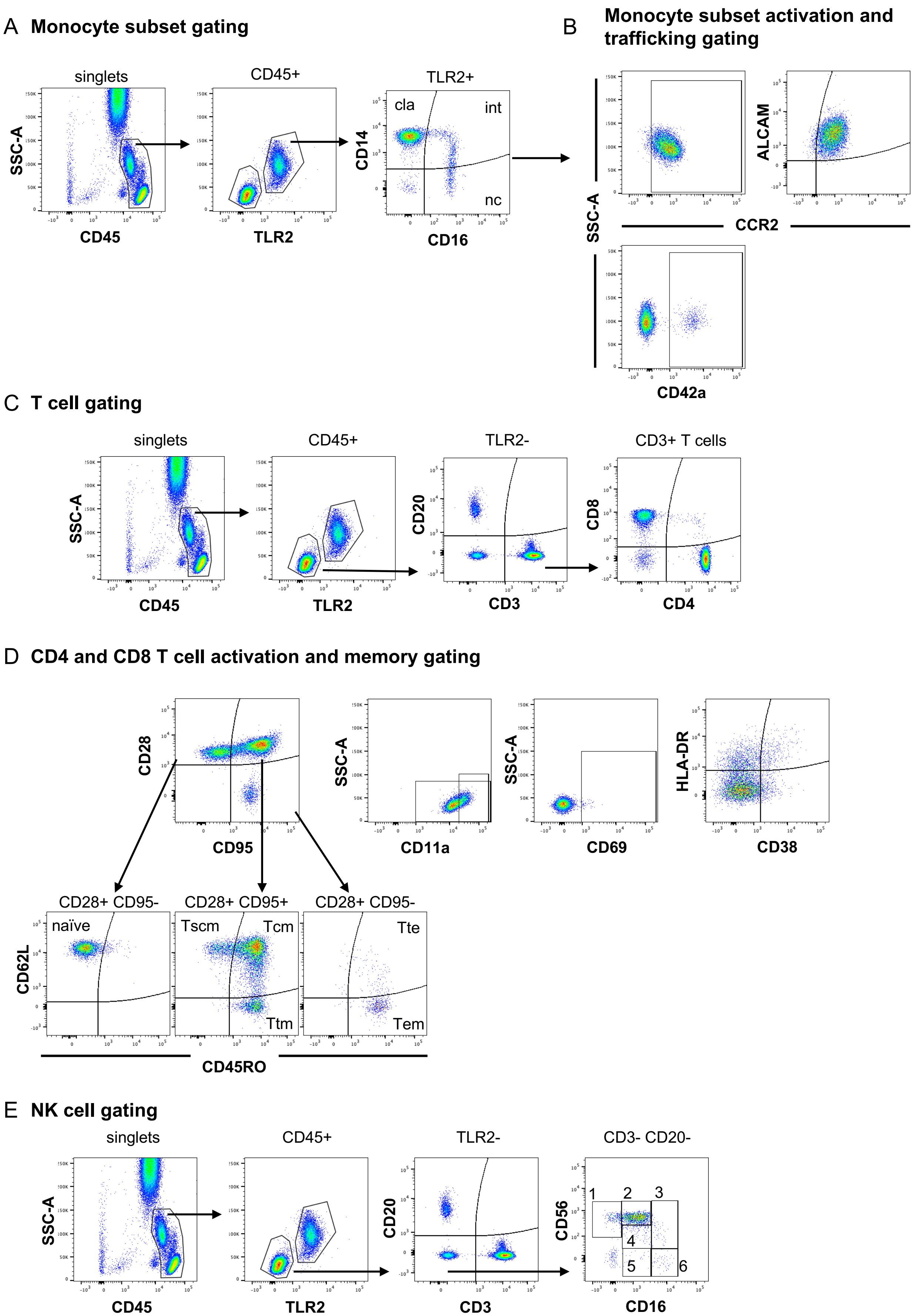

Supplemental Figure 9. Flow cytometry gating schemes. Monocyte subset gating, cla = classical, int = intermediate, nc = non-classical (A). Monocyte subset activation (CD42a) and trafficking (CCR2, ALCAM) gating (B). T cell gating (C) followed by CD4 and CD8 memory and activation (CD11a, CD69, CD38, HLA-DR) gating (D). NK cell gating, 1 = CD56brightCD16−, 2 = CD56brightCD16dim, 3 = CD56dim/CD16+, 4 = CD56dim/CD16dim, 5 = CD56dim/CD16-, 6 = CD56-/CD16+ (E).
